# Forensic Feature Exploration and Comprehensive Genetic Insights Into Yugu Ethnic Minority and Northern Han Population *via* a Novel NGS-Based Marker Set

**DOI:** 10.3389/fgene.2022.816737

**Published:** 2022-04-27

**Authors:** Qiong Lan, Congying Zhao, Chong Chen, Hui Xu, Yating Fang, Hongbing Yao, Bofeng Zhu

**Affiliations:** ^1^ Guangzhou Key Laboratory of Forensic Multi-Omics for Precision Identification, School of Forensic Medicine, Southern Medical University, Guangzhou, China; ^2^ Microbiome Medicine Center, Department of Laboratory Medicine, Zhujiang Hospital, Southern Medical University, Guangzhou, China; ^3^ Clinical Research Center of Shaanxi Province for Dental and Maxillofacial Diseases, College of Stomatology, Xi’an Jiaotong University, Xi’an, China; ^4^ Belt and Road Research Center for Forensic Molecular Anthropology Gansu University of Political Science and Law, Lanzhou, China

**Keywords:** NGS, forensic features, Yugu ethnic minority, genetic background, maternal lineages

## Abstract

The MPS technology has expanded the potential applications of DNA markers and increased the discrimination power of the targeted loci by taking variations in their flanking regions into consideration. Here, a collection of nuclear and extranuclear DNA markers (totally six kinds of nuclear genetic markers and mtDNA hypervariable region variations) were comprehensively and systematically assessed for polymorphism detections, further employed to dissect the population backgrounds in the Yugu ethnic group from Gansu province (Yugu) and Han population from the Inner Mongolia Autonomous Region (NMH) of China. The elevated efficiencies of the marker set in separating full sibling and challenging half sibling determination cases in parentage tests (iiSNPs), as well as predicting ancestry origins of unknown individuals from at least four continental populations (aiSNPs) and providing informative characteristic-related clues for Chinese populations (piSNPs) are highlighted in the present study. To sum up, different sets of DNA markers revealed sufficient effciencies to serve as promising tools in forensic applications. Genetic insights from the perspectives of autosomal DNA, Y chromosomal DNA, and mtDNA variations yielded that the Yugu ethnic group was genetically close related to the Han populations of the northern region. But we admit that more reference populations (like Mongolian, Tibetan, Hui, and Tu) should be incorporated to gain a refined genetic background landscape of the Yugu group in future studies.

## Introduction

From the perspective of forensic genetics, DNA analysis is the fundamental and core content and has become the cornerstone of this field for over 30 years since the first application of DNA fingerprinting in a paternity test case (Jeffreys, 2005). Nowadays, routine cases can be unquestionably solved by short tandem repeats (STRs)–based DNA profiling with the capillary electrophoresis (CE) genotyping platform. Autosomal STRs (A-STRs) are considered the gold standard for human identification and parentage testing. X- and Y-chromosomal STRs (X-STRs and Y-STRs) have served as efficient tools for identifying female and male pedigrees in complex kinship determination cases (Vermeulen et al., 2009; Ballantyne et al., 2010; Pinto et al., 2011; Kayser, 2017; Tillmar et al., 2017; Roewer et al., 2020). Particularly, due to the unique social and historical characteristics of Chinese families, Y-STR profiling has been proved to be extremely successful in solving cold cases, which on the other hand, also facilitates the general establishment of Y-STR databases (Ge et al., 2013). In general, STR-based analyses possess the superiorities of convenience and accuracy, which generate a standardized, quantitative method with strong statistical underpinnings and sufficient discrimination power in the criminal justice system.

Despite the substantial progress made in STR genotyping technologies, the frequent occurrences of challenging DNA from highly degraded, mixed, or trace samples force the forensic scientists to seek additional resolutions for difficult cases like criminal investigations, complex kinship determination, and disaster victim identifications (Oldoni and Podini, 2019; van Oorschot et al., 2019; Jin et al., 2020; Oldoni et al., 2020; Jin et al., 2021). DNA-based analysis provides informative clues for guiding the investigative direction and narrowing detective scope for investigators. Recently, single nucleotide polymorphism (SNP) has been regarded as a promising genetic marker because of the characteristics of genome abundance, low mutation rate, and applicability to individual identification, ancestry inference, lineage, and phenotype predictions (Abecasis et al., 2010). Well-constructed panels composed of ancestry-informative or phenotype-informative SNPs (aiSNPs or piSNPs) give valuable insights to identify unknown individuals (Walsh et al., 2013; de la Puente et al., 2016; Chaitanya et al., 2018; Lan et al., 2020). The mitochondrial DNA (mtDNA), which is inherited from maternal lineages, possesses properties of simple structure, low recombination rate, and abundance in the copy number. Knowledge about mtDNA phylogeny is essential for performing analysis on evolutionary anthropology, archaeology, medical genetics, and forensic genetics (Taylor and Turnbull, 2005; Kang et al., 2013; Blau et al., 2014; Zhang et al., 2016). As the second genetic legacy, except for nuclear DNA, mtDNA fulfills the demand of profiling fingernails, hair, and poorly degraded samples, thus making the mtDNA a suitable supplementary tool for forensic applications (Xu et al., 2019).

Massively parallel sequencing (MPS), also referring to the next-generation sequencing, yields extraordinary potential since its first official report in 2005 (Rogers and Venter, 2005). Since then, globally cooperative projects have progressively been launched to disclose life code from a genome perspective (Abecasis et al., 2010; Mallick et al., 2016). The superior advantages of high throughput and workflow efficiency drive MPS to be a powerful tool in life science. For forensic analysis, the vast majority of DNA markers can be co-analyzed and abundant genotypic data can be obtained by MPS within a short time, which compensates for the limitations of the CE method. Till now, different sequencing platforms are publicly available to researchers. The Illumina sequencer and the Ion Torrent sequencing platform are the two most representative sequencing platforms, with the former adopting laser to capture different bases and the latter using a semi-conductor device to monitor base changes, respectively (Børsting and Morling, 2015; Ravi et al., 2018). Currently, several multiplex amplification systems have been constructed based on different sequencing platforms in forensic science. The MiSeq FGx™ Forensic Genomics System (MiSeq FGx System) developed by the Illumina company is well received as a complete sample-to-profile MPS workflow for forensic DNA analysis. By multiplex amplifying over 200 forensically relevant genetic markers in one single reaction, the MiSeq FGx System fully displays the advantages of MPS technology and perfects the usage of diverse DNA markers for forensic applications (Xavier and Parson, 2017). However, it is noticeable that mtDNA variations have not been incorporated into the panel designation, which leads to the loss of maternal lineage information. Here, we introduce a novel assay simultaneously analyzing a total of 356 forensically relevant genetic markers and three mtDNA hypervariable regions in the present study. Compared with the MiSeq FGx System, more A-STRs (from 27 to 54), X-STRs (from 7 to 27), Y-STRs (from 24 to 48), iiSNPs (from 94 to 145), aiSNPs, piSNPs (from 78 to 82), and mtDNA were included into the panel construction for possessing higher polymorphisms in the Chinese populations or being more informative to predict ancestry or phenotype characteristics of Chinese.

As previously reported, patterns of human population structure are differentiated. The geographic distance creates certain constraint on the random mating of human populations. Therefore, the population structure in early human groups established as they continued to mate with immediate neighbors with whom they shared their ancestry. Pre-genomics studies of population variation have attempted to measure what structure exists in modern human populations using limited types of polymorphic markers, which on the other hand, favor the case–control studies in medical genetics and ancestry inference analysis in forensic genetics to avoid potentially erroneous estimations.

The Yugu group is one of the specialized ethnic minorities in Gansu province with a small population but a long history. It is suggested that the Yugu group is typically divided into three linguistically different clusters. The three languages are the east Yugu language (Altaic language family), west Yugu language (Mongolian language family), and Chinese, among which the Chinese language is used as an official language interchange tool for different tribes. The Yugu people still retain the old nomadic lifestyle of their ancestors but have also absorbed some living habits from the Han population (Zhao and Lin, 2020). Despite the well-recorded historical documents of the Yugu group, little is known about the genetic architecture within the contemporary Yugu group or the genetic influence on its gene pool made by other populations. Besides, the Han population is the most populous group in China and the Han Chinese are widely distributed throughout the whole country. Owing to the mixed residence of the Han population and the neighboring ethnic groups, gene interactions are prone to occur, and their genetic landscapes are undoubtedly influenced by each other. To assess the forensic efficiency of the novel system in the Chinese populations and to further obtain a comprehensive genetic background of the rarely focused Yugu group and the neighboring Han population, the Yugu and Han individuals are carefully analyzed in this study. The genetic variations of the analyzed populations along with previously published genotype data allow us to better describe their genetic legacy and explore their genetic relationships with other populations. Moreover, this is the first study to discuss the genetic architecture of the Yugu group from the genomic perspective.

## Materials and Methods

### Sample Collection

The Yugu group from the Gansu province and the Han population from the Inner Mongol Autonomous Region of China (NMH) were recruited into the current study as research subjects. The following standards were complied with by the participants: 1) self-reported healthy conditions; 2) no biological kinships related with anteriorly recruited objects within at least three generations; and 3) no immigration and intermarriage events in their family histories. Ultimately, a total of 165 Yugu and 333 NMH peripheral blood samples were collected. The storage condition of the blood samples was −20°C before DNA extraction. For longer preservation time, fresh blood samples were spread on FTA cards and dried at room temperature.

### Ethical Statement

Written informed consents were obtained from the participants before the experiments started. The study was conducted with the approval of the Ethics Committees of the Southern Medical University, Guangzhou, China, and the Xi’an Jiaotong University, Xi’an, China (No. XJTULAC201).

### DNA Extraction and Library Preparation

The genomic DNA of all the samples were isolated from bloodstains on the FTA cards with QIAmp DNA Blood Mini Kits (Qiagen, Hilden, Germany). By using a Qubit dsDNA HS Assay Kit (Thermo Fisher Scientific, San Francisco, CA, United States), the extracted DNA was subsequently quantified with Qubit 3.0 following the manufacture’s protocol. DNA libraries were prepared with a two-step PCR procedure by using the MGIEasy Identification system (MGI Tech, Shenzhen, China). The first-round PCR mix included 12.5 ul Enzyme Mix and 6 ul Primer pool. The thermal cycling conditions were programmed as follows: the initial incubation step was set at 98°C for 5 min, followed by 14 cycles of denaturation at 98°C for 15 s, annealing at 64°C for 1 min and 60°C for 1 min, and extension at 72°C for 30 s, as well as a final extension at 72°C for 2 min. The second-round PCR mix was set at a total volume of 19.5 ul, which included 12.5 ul Enzyme Mix, 3ul Primer Block, 2 ul PCR Dual Barcode Primer F, and 2 ul PCR Dual Barcode Primer R. The thermal cycling condition for second-round PCR was basically the same as the first round, except for the 16 cycles of denaturation at 98°C for 15 s, annealing at 64°C for 30 s and 60°C for 30 s, and extension at 72°C for 30 s. DNA Clean Beads (DNBs) (MGI Tech, Shenzhen, China) and Quant-iT PicoGreen dsDNA Assay Kits (Thermo Fisher Scientific, San Francisco, CA, United States) were used to purify and quantify DNA libraries, respectively. Then, the DNBs were generated by pooling the DNA libraries during the RCA procedure. Finally, MGISEQ-2000RS (MGI Tech, Shenzhen, China) was used to perform sequencing.

The raw sequencing data were subsequently analyzed for different genetic markers by following strict criteria. Detailed information concerning the procedures of data processing and genotype calling for different genetic markers was summarized in the Supplementary Material.

### Statistical Analyses

Statistical analyses were separately conducted in relation to different kinds of DNA markers. Prior to performing the various analyses, we evaluated the linkage disequilibrium (LD) status of pairwise loci to eliminate the impact of LD phenomenon on downstream analyses. We used the STRAF (http://cmpg.unibe.ch/shiny/STRAF/) to calculate descriptive parameters like gene diversity (GD), match probability (MP), power of discrimination (PD), probability of exclusion (PE), polymorphism information content (PIC), and observed heterozygosities (Hobs) to assess forensic efficiencies of the 54 A-STRs (Gouy and Zieger, 2017). A violin plot of forensic parameters (GD, Hobs, PD, and PE) for 54 A-STRs in Yugu and NMH populations was visualized by the *R* program. Single-locus Hardy–Weinberg equilibrium (HWE) tests and pairwise loci LD analyses were conducted by the command-based Genepop software (Rousset, 2008). The cumulative power of discrimination (CPD) and the combined probability of exclusion (CPE) were calculated with Excel by referring to the formula. The differences of observed alleles by CE and MPS methods were estimated by direct counting.

For Y-STRs, the haplotype frequencies were determined by the direct counting method. The GD for each locus was calculated according to *Nei* and Tajima. The haplotype diversity (HD) was calculated by referring to the formula: $HD=n\left(1-\sum_{i=1}^{n}p_{i}^{2}\right)/\left(n-1\right)$ , where n and *p*_*i*_ were the sample size and frequency of the *i*th haplotype, respectively.

Statistical analyses for X-STRs were separately conducted in male and female samples. Using the PowerStats v1.2 program (Promega, Madison, WI, United States), we estimated the allele frequencies of the 27 X-STRs in female samples. The Arlequin software (v.3.01) was used to calculate the allele frequencies in male samples (Excoffier et al., 2007). The differences in allelic frequencies among males and females were assessed by the standard analysis of variance (ANOVA) method using SPSS Version 13.0 software (SPSS Inc., Chicago, IL). A set of parameters, namely, PIC, expected heterozygosity (He), PE, power of discrimination in females (PDF), power of discrimination in males (PDM), mean exclusion chances (MECs) for deficiency cases, normal trios, and duo cases, were calculated using the function programmed in the online ChrX-STR.org 2.0 database (http://www.chrxstr.org). The LD tests of pairwise X-STR loci were analyzed in the Yugu and NMH populations using the Genepop v4.0.10.

The MGI system originally incorporated 145 highly polymorphic iiSNPs in the panel designation. After sequencing, 12 iiSNPs were removed from further analyses because of significant deviations from the HWE, which attributed to the nonspecific amplification of the 12 iiSNPs during PCR. Deeper inspection of the phenomenon revealed that highly homologous sequences were detected in the flanking regions of the 12 excluded iiSNPS. Forensic descriptive parameters for the rest of the 133 iiSNPs in Yugu and NMH populations were also calculated by STRAF and further visualized through TBtools (Chen et al., 2020a). After excluding the potential LD status of pairwise iiSNPs, the combined match probabilities (CMPs) in the Yugu group and NMH population were calculated with Excel by referring to the formula. A scatter plot exhibiting the decline tendency of the CMPs with the increased number of iiSNPs was generated by the *R* program with ggplot2 package. To further assess the statistical power of iiSNPs set for analyzing complex kinship testing scenarios, pairwise relationship tests including full siblings and half siblings were simulated by Familias v3 (Kling et al., 2014). We ran the simulations with two sets of DNA markers: the 54 A-STRs and the combination of 54 A-STRs and 133 iiSNPs. Distributions of likelihood ratios (LRs) for each kinship hypothesis (H1) versus the values for the unrelated hypothesis (H2) were calculated and assessed in the Yugu group and NMH population. The allele frequencies of the 54 A-STRs and 133 iiSNPs in the Yugu and NMH populations were used to simulate genotype data of each 1,000 full sibling and half sibling cases to generate LR distributions. During the simulation, we determined the recombination ratio of pairwise loci by referring to data reported by Li et al. (2021). LR distributions of the Yugu and NMH populations were plotted with the density plot function in the *R* program.

Haplogroup status for the analyzed Yugu and NMH individuals was assigned using HaploGrep 2 (https://haplogrep.i-med.ac.at) (Weissensteiner et al., 2016) based on PhyloTree build 17 (http://www.phylotree.org/index.htm) (Dür et al., 2021). Detailed haplogroup distributions of the overall 498 samples are listed in the Supplementary Material of mtDNA. For further analysis, we calculated the haplogroup frequencies by direct counting. A collection of statistical indexes, incorporating haplotype diversity (Hd), nucleotide diversity (pi), the number of segregating sites (s), and mismatch distributions with model test statistics (the sum of squared deviations, SSD); Harpending’s raggedness index (HRI), neutrality tests (Tajima’s D/Fu and Li’s F statistics), and the average number of pairwise nucleotide differences (k) were estimated using DnaSP v5 (Librado and Rozas, 2009).

### Population Genetic Analysis

Presently generated data of the Yugu and NMH populations and the genotype data of the reference populations were integrated to perform population genetic analysis. Detailed information of the reference populations for different genetic markers are summarized in the Supplementary Material. For Y-STR analysis, populations from the continents Africa, Central Asia, East Asia, West Asia, Europe, Oceania, and South America were labeled as referenced populations. Pairwise *F_ST_* among different populations were estimated by Arlequin v3.6.1 software. Then, multiscale dimensional (MDS) analyses were performed to reveal the spatial distributions of the studied populations and the referenced populations at seven continental population level and East Asian population level, respectively. Phylogenetic construction of the overall populations was generated based on population pairwise *F_ST_* with the neighbor-joining method by MEGA v6.06. Subsequent tree visualization and management were implemented by uploading the Newick tree file generated by MEGA to Evolview v2 (He et al., 2016).

SNP-based analyses were divided into three parts, namely, verification of the iiSNPs for personal identification and kinship determination (which was earlier stated) and the evaluation of aiSNPs for ancestry inference. Besides, phenotype estimation results with piSNPs were directly downloaded from the inbuilt software and is presented in the Supplementary Material of piSNPs. The populations from the 1000 Genomes Project were incorporated as comparison populations in the aiSNPs-based analyses. The principal component analysis (PCA) of the studied populations and comparison populations were performed at the individual level using the *R* program to evaluate the effectiveness of the aiSNPs for distinguishing unknown individuals from different populations and to determine the major factors accounting for the population genotype discrepancies. We adopted a stepwise method to generate clustering patterns of PCA from five to three continents. To deeply investigate the substructure patterns for the Yugu group and NMH population and to further infer their proportions of different ancestral genetic components, STRUCTURE v2.3.4 was used to perform unsupervised ancestral component prediction of the populations with the length of burn-in period 10,000 times followed by 10,000 MCMC repetitions (Pritchard et al., 2000). Based on the admixture model and allele frequencies correlated pattern, initial runs were performed without any prior information about the sample origins. Hypothetical ancestry clusters (*K*) were set from 2 to 7, with 15 replicates performed for each of the testing *K* values. The optimal *K* was identified with the online web Harvester program (http://taylor0.biology.ucla.edu/structureHarvester/). The average permutated individual and population Q-matrices for 15 replicates of each *K* were assessed by CLUMPP version 1.1.2 software (Jakobsson and Rosenberg, 2007), and subsequent plotting (the bar plot) was performed using DISTRUCT version 1.1 software with the input of CLUMPP results. A triangle plot was directly generated by STRUCTURE to reflect the cluster pattern of the analyzed populations and the reference populations from Africa, East Asia, and Europe.

To better visualize the discrepancies of haplogroup distributions among the two studied populations and the referenced populations, we uniformed the haplogroup frequencies by calculating the Z score values and further constructed a heat map plot by assigning colors to the corresponding haplogroups of different populations according to the estimated Z scores (Li et al., 2019). The PCA of all populations was plotted based on haplogroup frequencies by PAST v2 software. Major factors contributing to the PCA clustering pattern of different populations in the first two principal components and scree plot were also exported by PAST. To disclose the genetic affinities of the Yugu, NMH populations and the reference populations from the maternal aspect, *F*_*ST*_ values of pairwise populations were calculated based on the haplogroup frequencies with Arlequin software. Besides, to further intuitively display *F*_*ST*_ between the analyzed populations and their comparison populations, bar plots were constructed through the *R* program. Phylogenetic networks and trees of the mtDNA haplogroups in the Yugu group as well as the NMH population and the reference populations from East Asia were generated by Network v5.0 (https://www.fluxus-engineering.com/), and the plots were subsequently visualized in the Network Publisher (http://www.fluxus-engineering.com/index.htm). Bayesian skyline plots (BSP) for effective population size (Nef) through time in the Yugu and NMH populations were reconstructed using BEAST based on three hypervariable region sequences of the mtDNA, as described elsewhere (Drummond and Rambaut, 2007). In the BSP analysis, a strict molecular clock with a fixed rate of 3.02E-7 substitutions per site per year for mtDNA control region was selected according to a previous report (Endicott and Ho, 2008). Each Markov chain Monte Carlo simulation was run for 40,000,000 generations for Yugu and 160,000,000 generations for NMH, and sampled every 2,000 generations, with the first 0.1% of the total generations discarded as burn-in, and the subsequent results were visualized with Tracer v1.5.

## Results

### Massively Parallel Sequencing Performance

MPS performance metrics for the novel detection system were evaluated here to provide a reference for MPS applications, which included depth of coverage (DoC) and allele coverage ratio (ACR). In A-STRs, the DoCs were observed in the range of 583 ± 463× at SE33 to 13100 ± 5018× at D14S1434, with an averaged DoC of 3440 ± 2341×. The loci with a lower DoC (<1000×) were found at SE33, D14S608, D4S2408, D13S325, D6S1017, and D9S925 loci. For the 48 Y-STRs, the DoCs ranged from 148 ± 95× at DYS389II to 4792 ± 1996× at DYS645, with a average DoC of 1708 ± 1064×. Five Y-STR loci (DYS389II, DYS448, DYS518, DYS449, and DYS627) were discovered to have DoCs <500×. In the 27 X-STRs, DoC distributions were separately analyzed in the female and male samples. In females, the DoCs spanned from 774 ± 259× at DXS10079 to 13829 ± 5077× at DXS7423 with an average DoC of 4310 ± 3210×, while in males, the DoCs were in the range of 371 ± 147× at DXS10079 to 7448 ± 3151× at DXS7423 and the average DoC was 2110 ± 1640×. It was indicated that the DoCs of the females were basically twice the DoCs of the males. Three hypervariable regions of mtDNA were amplified with seven segments, and the Docs were varied from 1964× to 24577×. The averaged DoC was calculated to be 8621×. The detailed information concerning DoCs of the genetic markers is listed in the Supplementary Material.

The ACR was widely accepted to reflect imbalanced intra-locus signals which were created by stochastic effects in PCR. The distribution of ACRs in the Yugu and NMH populations was visualized by a boxplot. As shown in Supplementary Figure S1, the STRs were arranged in the ascending order according to the median value of the ACRs at each STR locus. It indicated that the medians for almost all the loci excessed the ACR threshold of 0.5 (except D12ATA63 in Yugu and NMH) with 91% of the loci displaying the ACR medians close to or greater than the ACR threshold of 0.75.

### Sequencing Variations Detected in the Short Tandem Repeat Motif

The population data from the 1000 Genomes Project were used to detect the theoretically existing variants in the motif and flanking regions of the analyzed STRs and SNPs. Among the analyzed iiSNPs, 23 different variants were investigated within their primer binding region with minor allele frequencies (MAFs) >0.1. Eight tri-allelic SNPs were detected adjacent to seven SNPs (rs5745448, rs11123823, rs6955448, rs200354, rs2342747, rs430046, and rs1657741). Eighty-four SNPs were discovered in the flanking regions across the 32 A-STR loci spreading over 17 chromosomes. The increased variants were most frequently observed in the D5S2800 locus (14 variants), followed by the D2S441 locus (6 variants). Detailed information concerning the additional variants detected in the motif and flanking regions of the population data from the 1000 Genomes Project is listed in the Supplementary Material of A-STRs.

We further investigated the increased variants in the sequences of A-STRs in the studied Yugu group and NMH population by comparing the observed alleles of length-based (LB) polymorphisms and sequence-based (SB) polymorphisms. We discuss the allele increment in the repeat region of the targeted STR loci here. As shown in Supplementary Figures S2A,B, the fragment alleles and additional alleles increased by variations in the STR motif were labeled in different colors for the Yugu group and NMH population, respectively. And the corresponding increment of each locus was represented by red triangles or red rhombus. In the Yugu group, the results indicated that the most significant increments were observed at D8S1179, D7S3048, and D21S11 loci, with a detectable allele gain percentage of 54% (29). While in NMH population, additional variations were found to more frequently occur at D11S4463, D13S317, and D8S1132 loci, and the observable allele gain percentage was 59% (32). Similar patterns of allele increment (>50%) were detected at D8S1179, D7S3048, and D8S1132 loci in the two studied populations. We also discovered that several loci exhibited no appreciable allele gains in the Yugu and NMH populations; see detailed information of the unique alleles observed by LB polymorphisms and SB polymorphisms in the Supplementary Material.

### Forensic Descriptive Parameters for Different Classes of DNA Markers

#### Genetic Polymorphisms of Autosomal Short Tandem Repeats in Yugu and NMH Populations

HWE (*p* = 0.05/54 = 0.00093) and LD (*p* = 0.05/1431 = 0.00003) tests were performed before subsequent analyses, and the results showed that neither the single STR locus nor the pairwise STR loci exhibited significant deviations from equilibrium tests after Bonferroni’s correction. In this study, length-based allele frequencies and forensic descriptive parameters of the 54 A-STRs in Yugu and NMH were listed in the Supplementary Material. The collection of parameters included Hobs, PIC, GD, PD, PM, PE, and TPI. The Hobs ranged from 0.5697 at D1S1677 to 0.9455 at Penta E and 0.6078 at TPOX to 0.9461 at SE33, the PICs from 0.5145 (D1S1627) to 0.9329 (SE33) and 0.5480 (TPOX) to 0.9300 (SE33), the GDs from 0.5905 (D1S1627) to 0.9395 (SE33) and 0.6136 (TPOX) to 0.9354 (SE33), the PDs from 0.7330 (D1S1627) to 0.9877 (SE33) and 0.7803 (D1S1627) to 0.9884 (SE33), the PMs from 0.0123 (SE33) to 0.2670 (D1S1627) and 0.0116 (SE33) to 0.2197 (D1S1627), and the PEs from 0.2561 (D1S1677) to 0.8889 (Penta E) and 0.3026 (TPOX) to 0.8960 (SE33) in the Yugu group and NMH population, respectively.

**FIGURE 1:**
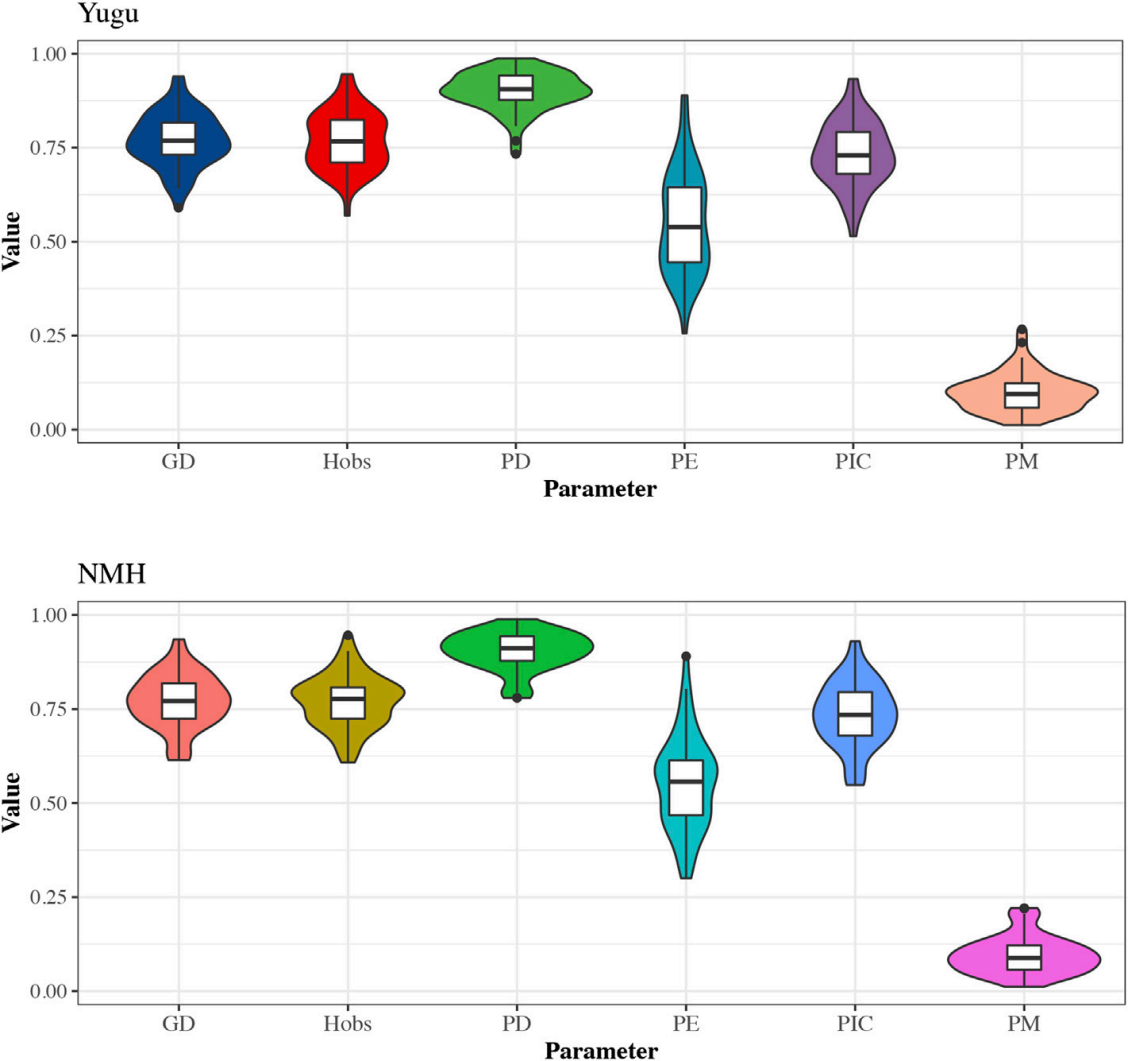
The violin plot of the forensic descriptive parameters for 54 A-STRs in the Yugu and NMH populations. The upper and lower edges of the box represent the upper and lower quartiles of data, respectively, and the box contains 50% of the data. The scattered points outside the box represent outliers.

The forensic statistical parameters for the 54 A-STRs were also visualized through violin plots generated by the *R* program. As shown in Figure 1, the boxes labeled in different colors were shown to represent data dispersion of the parameters in Yugu and NMH populations, respectively. The median values of GDs, Hobs, PDs, and PICs were higher than or approximately close to 0.75 horizontal line in both Yugu and NMH populations. Even the median values of PDs were higher than 0.85 and barely close to 0.9, and the PEs were over 0.5 in the studied two populations, while the median values of PMs in Yugu and NMH were less than 0.12.

#### Genetic Diversities of Y Chromosomal Short Tandem Repeat Loci in These Two Populations

The distinct alleles and GDs of the 48 Y-STR loci in the Yugu and NMH populations are shown in the Supplementary Material. The Y-STR loci were divided into two groups: the single-copy allele group and the multi-copy allele group. In the single-copy allele group, a total of 253 and 248 distinct alleles were detected in the Yugu and NMH populations, respectively, and null alleles were more frequently observed in the NMH population at seven loci (DYS389II, DYS437, DYS439, DYS448, DYS458, DYS449, and DYS627) than in the Yugu group at two loci (DYS448 and DYS531). Six different microvariants were found separately at four loci (DYS645, DYS531, DYS505, and DYS518), of which four microvariants were found at DYS531 and DYS505 loci in the Yugu group. While in the NMH population, none of the alleles exhibited detectable microvariants in this study. Allele combinations and the corresponding frequencies for multi-copy loci DYS385a/b, DYF387S1, DYS527, DYS459, and DYF404S1 are also presented in Supplementary Material. Two multi-copy loci were most frequently observed, while tri-allelic combinations also emerged with rare frequencies at two multi-copy loci. It was indicated that microvariants were discovered in different loci in the Yugu (DYS527) group and NMH (DYS385) population. Besides, we also observed di-allelic combinations at different loci in the Yugu (DYS19 and DYS612) and NMH (DYS643, DYS518, DYS557, and DYS612) populations with the frequencies close to or less than 0.01.

A total of 89 and 159 unique haplotypes were observed in Yugu group and NMH population, respectively. The HDs of the 48 Y-STR loci were calculated to be 0.9898 and 0.9937 in the two studied populations, indicating their promising power in forensic applications. GDs of the analyzed Y-STR loci ranged from 0.2539 at DYS391 to 0.9390 at DYS527 in Yugu and from 0.0841 at DYS645 to 0.9542 at DYS385 in NMH, with five loci (10.42%) and three loci (6.25%) below 0.50 in the Yugu and NMH populations. The GDs were above 0.7 at 26 loci (54.17%) in Yugu and 20 loci (41.67%) in NMH. The highest GD was observed at DYS527 (0.9390) in Yugu and DYS385a/b (0.9542) at NMH. The rapid mutated Y-STR loci showed high GD values (>0.70) in the two populations.

#### Genetic Diversities of X Chromosomal Short Tandem Repeat Loci in These Two Populations

According to the HWE test results in the female samples, all X-STR loci showed no deviations from the HWE after Bonferroni correction (*p* > 0.05/27 = 0.0019). The least *p* values were found to be 0.0124 in Yugu and 0.0020 in NMH. The LD tests were performed for all pairs of X-STR loci in the two studied populations. Among the overall 351 comparisons, no significant LDs were observed for a significance level of 0.00014 (*p* = 0.05/351 = 0.00014) after Bonferroni correction in Yugu ethnic group. While in NMH, several pairwise X-STR loci showed deviations from linkage equilibrium.

Besides, no significant differences were detected among the allelic frequencies of the 27 X-STR loci between the male and female samples in the Yugu and NMH populations by ANOVA, which supported that these X-STR loci presented no gender bias of allele distributions in the two studied populations. So, we pooled the allele frequencies of the male and female samples together for further analysis. As indicated in the Supplementary Material, a total of 220 and 250 alleles were found at the 27 X-STR loci in Yugu and NMH populations, respectively. DXS10135 was found to be the most polymorphic locus in both Yugu and NMH populations. Detailed information concerning the allelic frequencies of the 27 X-STR loci in Yugu and NMH are presented in the Supplementary Material.

Forensic parameters of the 27 X-STRs in the studied Yugu and NMH populations are shown in Table 1. The PICs ranged from 0.3995 at DXS7133 to 0.9023 at DXS10135 in Yugu, while from 0.2913 at DXS6800 to 0.9072 at DXS10135 in NMH. Totally, 92.59% (25/27) and 88.89% (24/27) of the loci were informative (PIC >0.5) in Yugu and NMH, respectively. DXS10135 was found to be the most informative locus, followed by DXS10148 both in the Yugu and NMH populations. The He values spanned from 0.4343 at DXS7133 to 0.9092 at DXS10135 and from 0.3063 at DXS6800 to 0.9135 at DXS10135 in the Yugu and NMH populations. Among the 27 X-STR loci, DXS10135 showed the highest He values in the two studied populations. The PE values varied from 0.1362 at DXS10079 to 0.8143 at DXS7423 in Yugu, and from 0.0662 at DXS10162 to 0.8232 at DXS7423 in NMH. The PDF values ranged from 0.6452 at DXS7133 to 0.9849 at DXS10135 and from 0.5037 at DXS6800 to 0.9862 at DXS10135 in Yugu and NMH, respectively. The PDM values ranged from 0.4343 at DXS7133 to 0.9092 at DXS10135 in Yugu, and from 0.3063 at DXS6800 to 0.9135 at DXS10135 in NMH. The cumulated PDFs for the two populations were estimated to be 0.999999999999999999999999984 (Yugu) and 0.99999999999999999999999985 (NMH). And the combined PDMs were calculated to be 0.999999999999999665 and 0.999999999999999663 in the Yugu and NMH populations, respectively. Besides, the MECs for deficiency cases, normal trios and duo cases were represented by MEC_df_, MEC_t_, and MEC_d_. The MEC_df_ ranged from 0.2381 at DXS7133 to 0.8174 at DXS10135 in the Yugu and from 0.1677 at DXS6800 to 0.8258 at DXS10135 in the NMH population. The MEC_t_ spanned from 0.3995 at DXS7133 to 0.9023 at DXS10135 in the Yugu and from 0.2913 at DXS6800 to 0.9070 at DXS10135 in the NMH populations. The last MEC_d_ was in the range of 0.2627 at DXS7133 to 0.8289 at DXS10135 and 0.1775 at DXS6800 to 0.8366 at DXS10135 in the Yugu and NMH populations, respectively. The combined MECs for deficiency cases, normal trios, and duo cases were 0.9999999973, 0.999999999999987, and 0.99999999975 in the Yugu ethnic group, while 0.9999999976, 0.999999999999988, and 0.99999999978 in the NMH population, respectively.

**TABLE 1:** Forensic efficiency parameters of the 27 X-STR loci in the Yugu group and NMH population (N_*Yugu*_ = 165; N_*NMH*_ = 333).

Loci	PIC		He		PE		PDF		PDM		MEC_df_		MEC_t_		MEC_d_	
	Yugu	NMH	Yugu	NMH	Yugu	NMH	Yugu	NMH	Yugu	NMH	Yugu	NMH	Yugu	NMH	Yugu	NMH
DXS10074	0.7565	0.7482	0.7871	0.7812	0.5754	0.5646	0.9240	0.9192	0.7871	0.7812	0.5887	0.5761	0.7565	0.7482	0.6281	0.6179
DXS10103	0.7270	0.7367	0.7633	0.7713	0.5329	0.5469	0.9077	0.9131	0.7633	0.7713	0.5487	0.5607	0.7270	0.7367	0.5927	0.6039
DXS10135	0.9023	0.9072	0.9092	0.9135	0.8143	0.8232	0.9849	0.9862	0.9092	0.9135	0.8174	0.8258	0.9023	0.9070	0.8289	0.8366
DXS7132	0.6995	0.7138	0.7395	0.7536	0.4920	0.5159	0.8921	0.8995	0.7395	0.7536	0.5138	0.5292	0.6995	0.7138	0.5603	0.5768
DXS7423	0.5241	0.4433	0.5949	0.5252	0.2848	0.2105	0.7651	0.6927	0.5949	0.5252	0.3195	0.2535	0.5167	0.4433	0.3794	0.3049
DXS8378	0.5234	0.5503	0.5945	0.6127	0.2844	0.3064	0.7645	0.7876	0.5945	0.6127	0.3252	0.3510	0.5233	0.5503	0.3786	0.4032
HPRTB	0.6753	0.6633	0.7249	0.7106	0.4678	0.4448	0.8748	0.8690	0.7249	0.7106	0.4780	0.4698	0.6753	0.6633	0.5332	0.5203
DXS10075	0.6600	0.6604	0.7064	0.7062	0.4382	0.4379	0.8675	0.8678	0.7064	0.7062	0.4675	0.4707	0.6600	0.6604	0.5167	0.5178
DXS10079	0.7718	0.7745	0.7995	0.8017	0.5982	0.6022	0.9320	0.9335	0.7995	0.8017	0.6095	0.6144	0.7718	0.7745	0.6467	0.6506
DXS101	0.8004	0.7790	0.8225	0.8058	0.6414	0.6098	0.9464	0.9355	0.8225	0.8058	0.6534	0.6205	0.8004	0.7790	0.6840	0.6563
DXS10148	0.8914	0.8978	0.8997	0.9056	0.7948	0.8069	0.9817	0.9833	0.8997	0.9056	0.7987	0.8080	0.8914	0.8978	0.8121	0.8212
DXS10159	0.7463	0.7467	0.7801	0.7807	0.5627	0.5638	0.9179	0.9179	0.7801	0.7807	0.5721	0.5728	0.7463	0.7467	0.6153	0.6160
DXS10162	0.6790	0.7351	0.7245	0.7712	0.4672	0.5467	0.8786	0.9116	0.7245	0.7712	0.4878	0.5569	0.6790	0.7351	0.5378	0.6019
DXS10164	0.5520	0.5672	0.5908	0.6066	0.2800	0.2989	0.7937	0.8059	0.5908	0.6066	0.3690	0.3845	0.5520	0.5672	0.4031	0.4189
DXS6789	0.7563	0.7934	0.7886	0.8177	0.5780	0.6323	0.9230	0.9425	0.7886	0.8177	0.5846	0.6409	0.7563	0.7934	0.6271	0.6745
DXS6795	0.6648	0.6503	0.7065	0.6946	0.4383	0.4200	0.8722	0.8624	0.7065	0.6946	0.4772	0.4627	0.6648	0.6503	0.5218	0.5069
DXS6800	0.4430	0.2913	0.4682	0.3063	0.1612	0.0662	0.6919	0.5037	0.4682	0.3063	0.2809	0.1677	0.4430	0.2913	0.3004	0.1775
DXS6807	0.5805	0.6091	0.6462	0.6698	0.3500	0.3831	0.8091	0.8303	0.6462	0.6698	0.3805	0.4093	0.5805	0.6091	0.4353	0.4642
DXS6809	0.7990	0.7859	0.8231	0.8121	0.6426	0.6216	0.9446	0.9385	0.8231	0.8121	0.6470	0.6281	0.7990	0.7859	0.6812	0.6643
DXS6810	0.5744	0.5431	0.6412	0.6167	0.3433	0.3114	0.8044	0.7795	0.6412	0.6167	0.3675	0.3352	0.5744	0.5431	0.4273	0.3966
DXS7133	0.3995	0.3957	0.4343	0.4452	0.1362	0.1439	0.6452	0.6427	0.4343	0.4452	0.2381	0.2285	0.3995	0.3957	0.2627	0.2603
DXS981	0.7786	0.8193	0.8055	0.8383	0.6093	0.6720	0.9353	0.9549	0.8055	0.8383	0.6194	0.6811	0.7786	0.8193	0.6556	0.7087
DXS9902	0.6140	0.5838	0.6764	0.6488	0.3927	0.3535	0.8329	0.8117	0.6764	0.6488	0.4070	0.3768	0.6140	0.5838	0.4678	0.4366
DXS9907	0.5029	0.5469	0.5850	0.6197	0.2733	0.3152	0.7456	0.7826	0.5850	0.6197	0.3034	0.3480	0.5029	0.5469	0.3605	0.4028
GATA165B12	0.5839	0.5332	0.6367	0.5901	0.3372	0.2792	0.8153	0.7751	0.6367	0.5901	0.3857	0.3362	0.5839	0.5332	0.4355	0.3850
GATA172D05	0.7235	0.6944	0.7558	0.7331	0.5198	0.4814	0.9080	0.8901	0.7558	0.7331	0.5490	0.5108	0.7235	0.6944	0.5884	0.5549
GATA31E08	0.7141	0.7314	0.7539	0.7664	0.5165	0.5383	0.8996	0.9104	0.7539	0.7664	0.5305	0.5541	0.7141	0.7314	0.5776	0.5975

Note: PIC, polymorphic information content; He, expected heterozygosity; PE, probability of exclusion; PDF, power of discrimination in females; PDM, power of discrimination in males; MEC_df_, mean exclusion chances for deficiency cases; MEC_t_, mean exclusion chances for normal trios; MEC_d_, mean exclusion chances for duo cases.

### Population Genetic Analysis Based on Different DNA Markers

#### Y-STR Based Analysis

**FIGURE 2:**
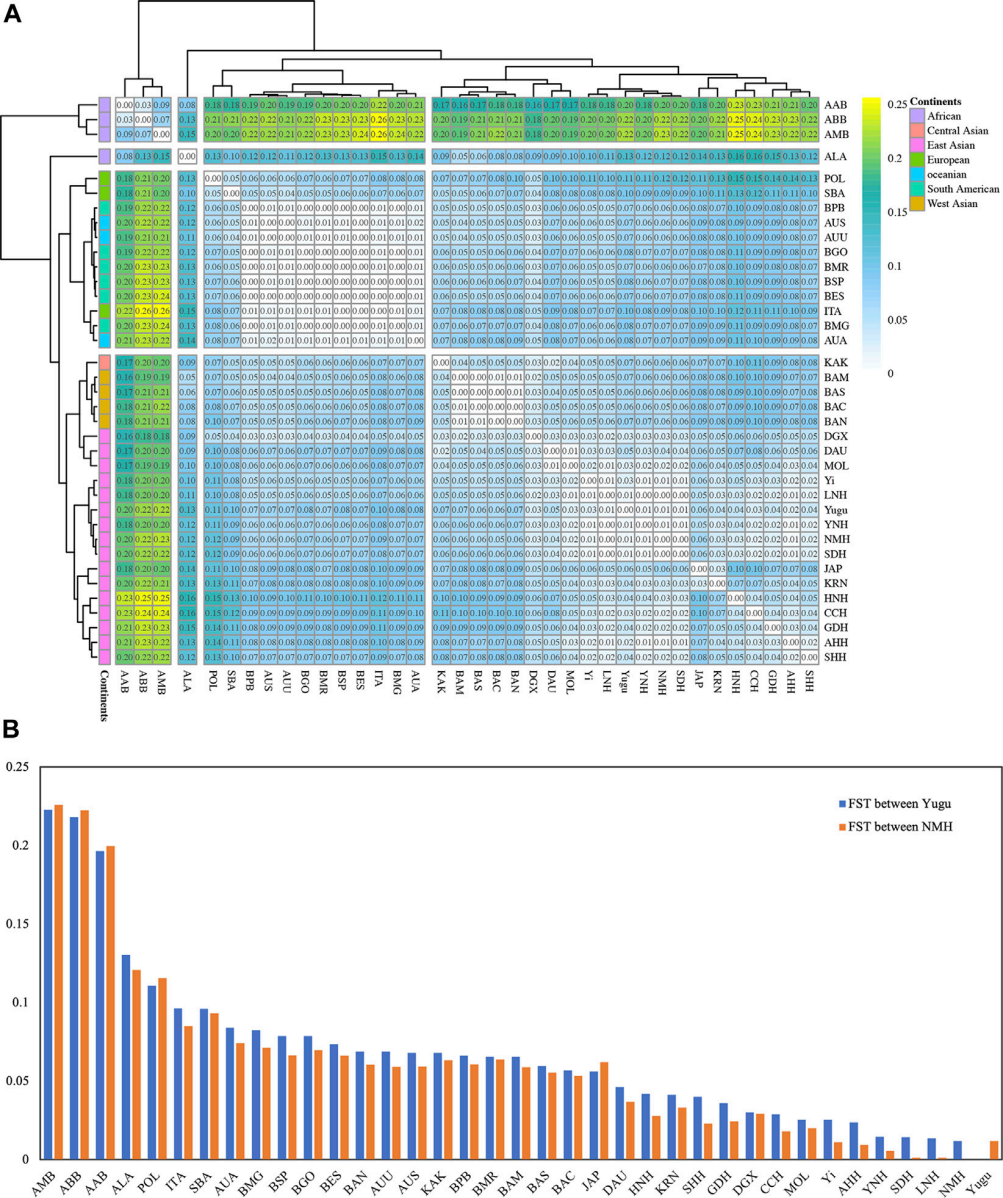
**(A)** Pairwise *F_ST_* among the two studied populations and the reference populations estimated based on the Y-STR genotype data. The color gradient for the *F_ST_* ranges from white to yellow, corresponding to the *F_ST_* from low to high. At the top and left sides of the heat map, clustering trees are generated based on the *F_ST_*. **(B)** Pairwise *F_ST_* between Yugu and the reference populations (blue bar), as well as NMH and the reference populations (orange bar).

In this part, referenced populations collected from worldwide regions were divided into seven groups to perform further analysis. In the first step, *F_ST_* were estimated among the analyzed populations and the referenced populations to show genetic differentiations of the overall populations based on the shared 27 Y-STR data available in the Y filer plus kit, then the *F_ST_* values were intuitively reflected by a heat map (Figure 2A). As expected, populations from the same continent shared smaller *F_ST_* (the grey to green color) and were basically positioned at the same sub-branch of the clustering tree at the bottom and left edge of the picture. Less genetic differentiations were detected between Yugu, NMH, and most East Asian populations, except for DAU and JAP populations which showed relatively larger *F_ST_* from the perspective of the Y STR-based analysis. The two studied populations exhibited the greatest degree of differentiations with the Africa populations, followed by Europe, South America, Oceania, West Asia, and Central Asia populations. Besides, the bar plot (Figure 2B) exhibiting the pairwise *F_ST_* between the two studied populations and the reference populations further indicated the closer genetic affinities among LNH, SDH, Yugu and NMH populations.

**FIGURE 3:**
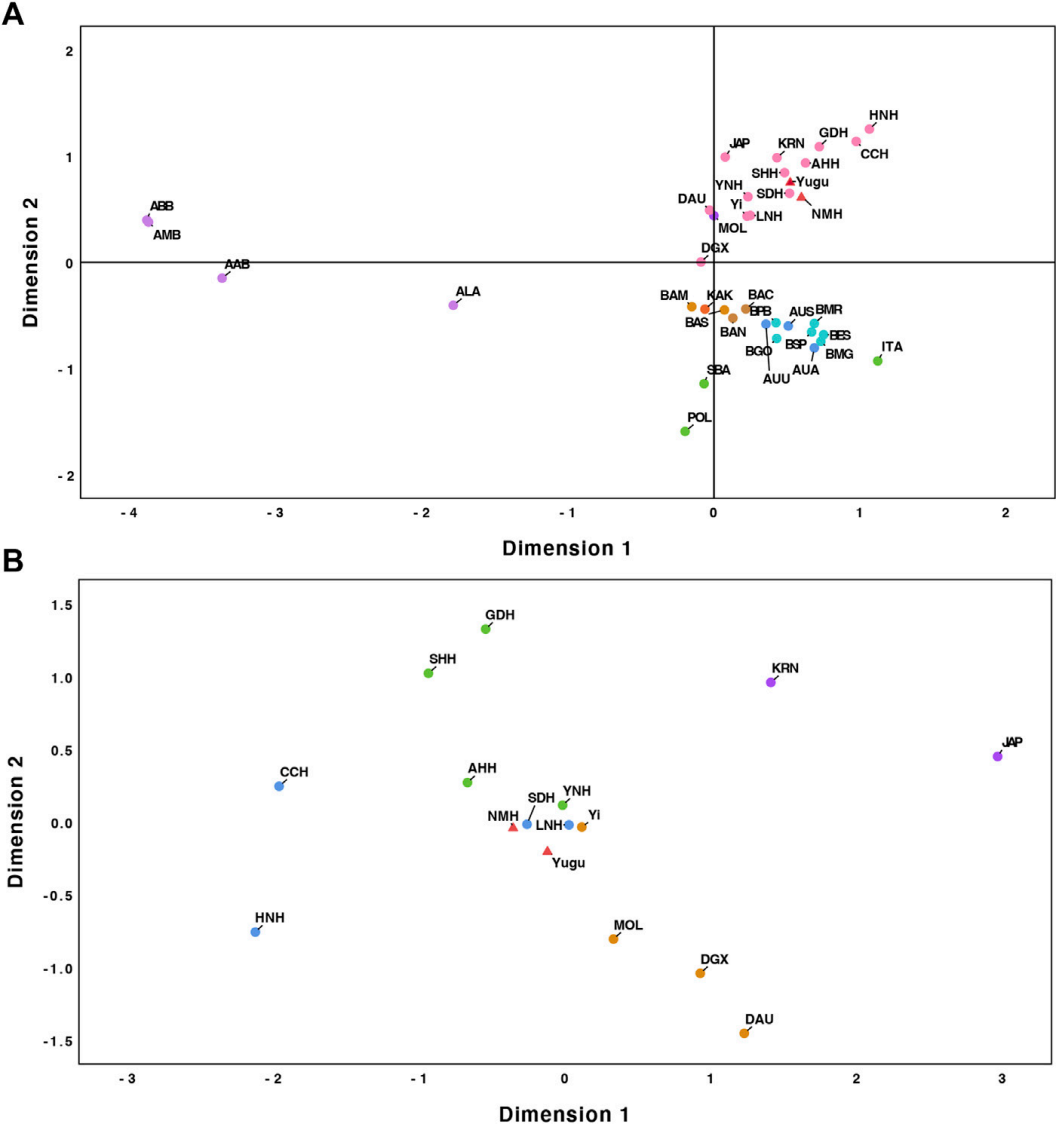
**(A)** MDS analysis of the overall 37 populations; **(B)** MDS analysis among the studied populations and the reference populations from East Asian.

Based on the pairwise *F_ST_*, we further conducted MDS analysis to provide an intuitive way to interpret patterns of divergence amongst the recruited population sets. The combined dimension 1 and dimension 2 were used to capture a large percentage of the total variation and efficiently represent the main pattern of genetic divergences exhibited in these populations. As indicated in Figure 3A, the East Asia populations were distinctively differentiated from the other continental populations and positioned at the upper right corner of the plot. South America, Oceania, and West Asia populations were not distinguishable through MDS analysis but showed relatively significant genetic differences with populations from the East Asian cluster. For better discrimination of genetic affinities among the populations from East Asia, we performed a refined MDS plot among the studied two populations and the referenced East Asia populations. As shown in Figure 3B, the populations approximately assembled according to their biogeographic locations. A distinct population cluster composed of NMH, SDH, LNH, Yi, and YNH populations was easily captured, which demonstrated that the Yugu group and the NMH population might share greater genetic similarities with the above-mentioned populations.

Subsequently, the circular phylogenetic tree coupled with pairwise *F*_*ST*_ values (Supplementary Figure S3) of the overall populations was constructed by executing programming codes using the online tool Evolview. Five primary branches could be easily distinguished: 1) the African branch consisted of ALA, AMB, AAB, and ABB populations; 2) the East Asian branch composed of HNH, CCH, AHH, GDH, SDH, YNH, SHH, LNM, Yi, KRN, JAP, DAU, MOL, and DGX populations and the studied Yugu and NMH populations; 3) the Oceania and South Asia branch comprising AUU, AUA, AUS and BMR, BPB, BGO, BMG, and BES populations; 4) the West Asia branch made up of BAC, BAM, BAN, and BAS populations; and 5) the European branch comprising POL and SBA populations. In general, each population clustered in accordance with its geographic location. Furthermore, pairwise *F*_*ST*_ between the two studied populations and the reference populations also gave insights into population genetic relationships. It was suggested that the genetic similarities were detected among populations in the same branch of the cluster tree, which meant that pairwise *F*_*ST*_ further supported the results of phylogenetic analysis.

### Single Nucleotide Polymorphism–Based Analysis

#### Forensic Efficiency Estimations of the iiSNPs

It was worthwhile to gain knowledge of the iiSNPs efficiencies for forensic purposes of personal identification and parentage testing. Altogether 133 iiSNPs were included in the following analysis. The forensic description parameters and the similarity clustering of the iiSNPs are shown in Supplementary Figure S4A (Yugu) and S4B (NMH), which included PE, Hobs, PD, PM, PIC, and GD. As indicated in Supplementary Figures S4A,B, the PDs of the iiSNPs in Yugu group (the green color) and NMH population (the purple color) exhibited considerable informativeness (over 0.7) among the analyzed six parameters, while efficiencies of the Hobs, PM, PIC, and GD were in the middle range. It was also pointed out that the iiSNPs which showed analogous efficiencies were intended to assemble in the same branch of the cluster tree.

The distribution of CPM (Supplementary Figure S5) was plotted in dash lines with the horizontal axis representing the number of loci and the vertical axis documenting the CPMs. The descending tendencies of CPMs in both Yugu (the green line) and NMH (the red line) populations were exhibited with the increasing number of iiSNPs. The estimated CPMs for sets of iiSNPs shared basically identical tendencies in the Yugu group and NMH population. The two intersectional dash lines in the graph indicated that less than 40 iiSNPs were capable of meeting the theoretical probability of individual identification.

Since the detection system was newly developed and launched for public use, we also performed analysis for forensic complex kinship determinations based on the incorporated STRs and iiSNPs. Likelihood distributions from relationship test simulations to evaluate the efficiency of the 54 A-STRs and 133 iiSNPs were shown for full sibling and half sibling cases.

**FIGURE 4:**
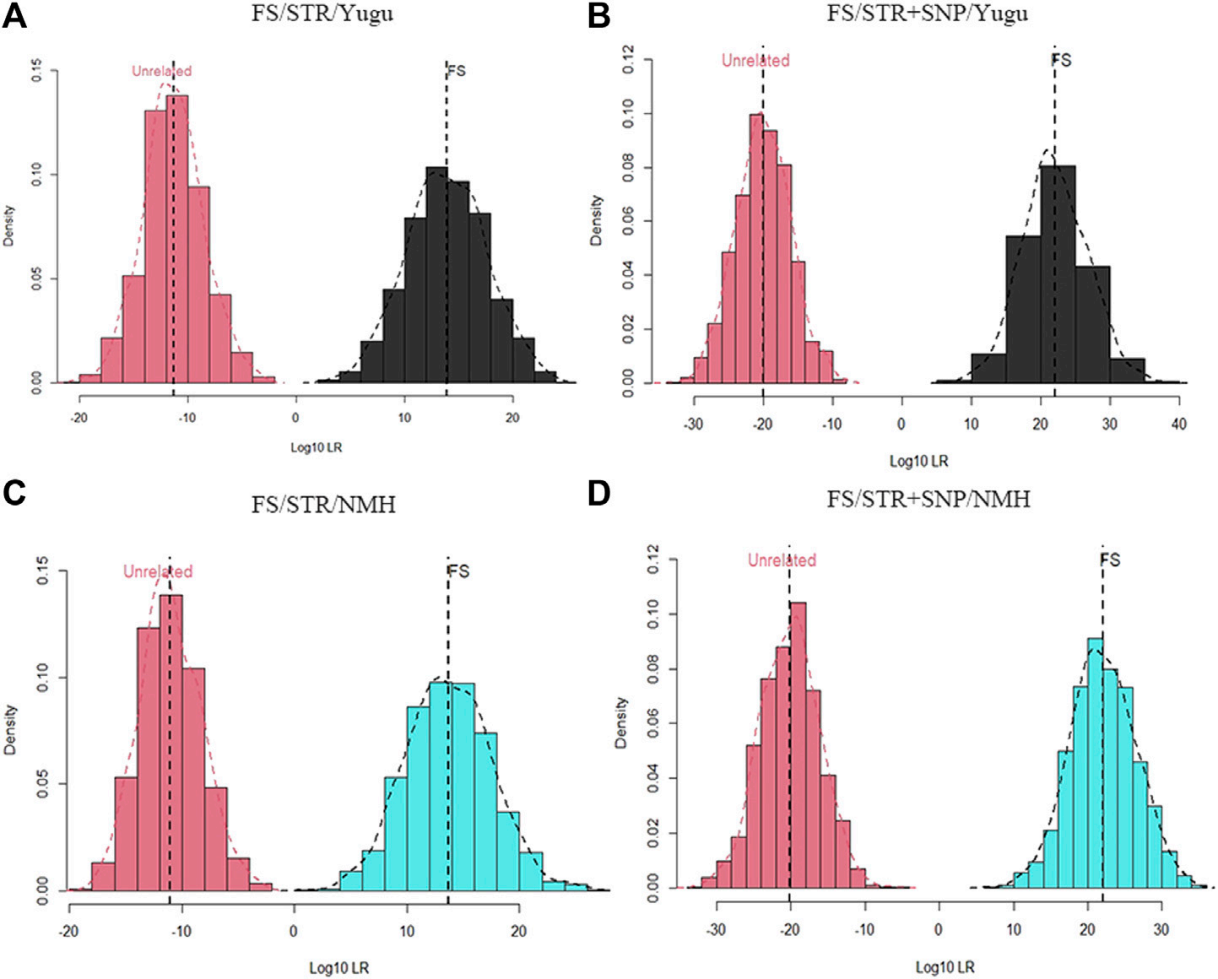
Log_10_LR distribution plots obtained from 1000 simulations of relationship tests between full siblings and unrelated individuals. **(A)** Log_10_LR distributions when only using 54 A-STR loci to distinguish full siblings from unrelated individuals in the Yugu ethnic group; **(B)** Log_10_LR distributions when using 54 A-STR loci and 133 iiSNPs to distinguish full siblings from unrelated individuals in the Yugu ethnic group; **(C)** Log_10_LR distributions when only using 54 A-STR loci to distinguish full siblings from unrelated individuals in the NMH population; **(D)** Log_10_LR distributions when using 54 A-STR loci and 133 iiSNPs to distinguish full siblings from unrelated individuals in the NMH population.

**FIGURE 5:**
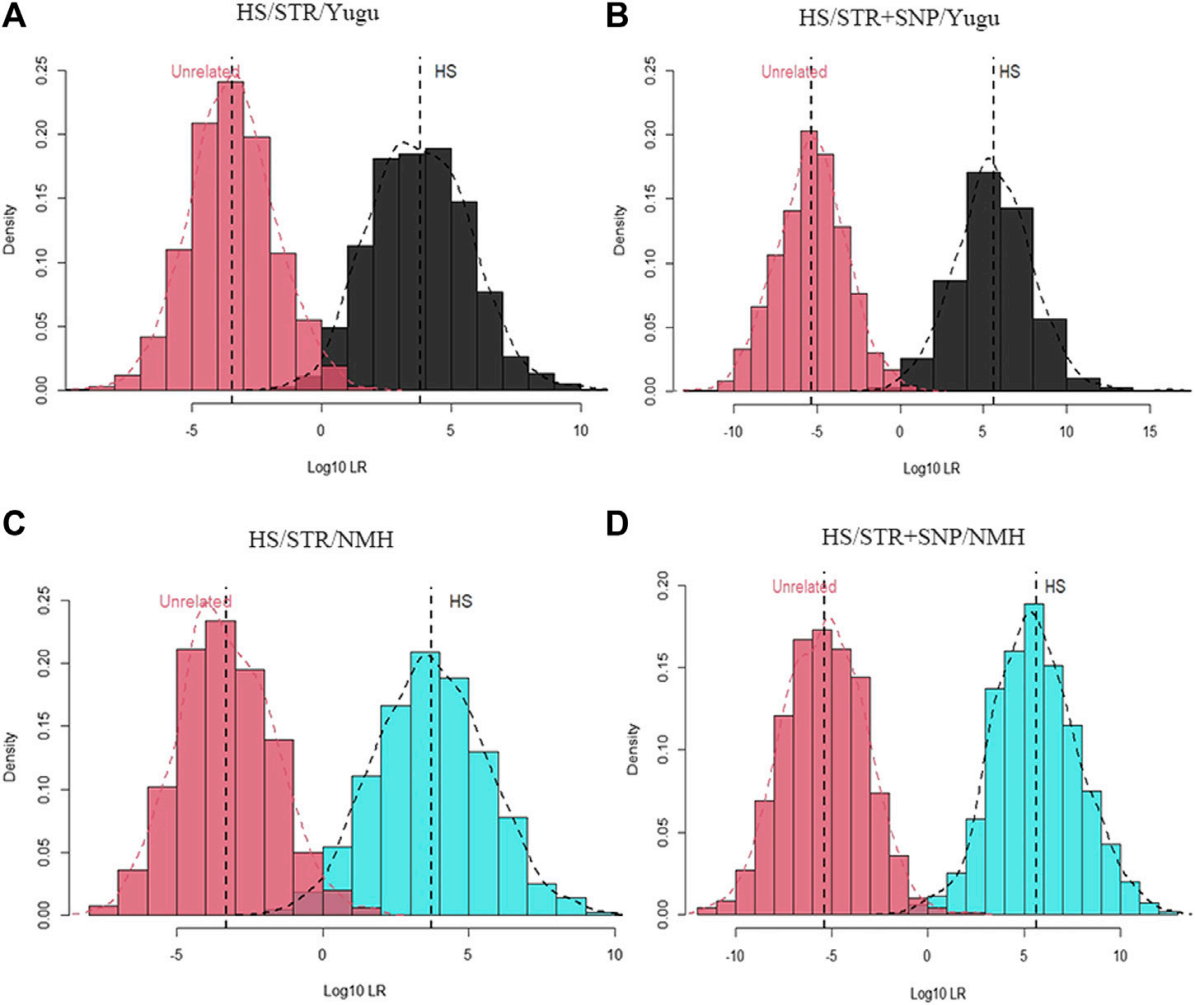
Log_10_LR distribution plots obtained from 1000 simulations of relationship tests between half siblings and unrelated individuals. **(A)** Log_10_LR distributions when only using 54 A-STR loci to distinguish half siblings from unrelated individuals in the Yugu ethnic group; **(B)** Log_10_LR distributions when using 54 A-STR loci and 133 iiSNPs to distinguish half siblings from unrelated individuals in the Yugu ethnic group; **(C)** Log_10_LR distributions when only using 54 A-STR loci to distinguish half siblings from unrelated individuals in the NMH population; **(D)** Log_10_LR distributions when using 54 A-STR loci and 133 iiSNPs to distinguish half siblings from unrelated individuals in the NMH population.

As shown in Figure 4 and Figure 5, the combination of the A-STR and iiSNP data sets as well as the individual STR set were separately tested for full sibling and half sibling determination cases in the Yugu and NMH populations. For full sibling tests, the likelihood distributions clearly separated full siblings and unrelated individuals either with A-STRs or a combination of the A-STRs and iiSNPs sets in both the Yugu and NMH populations, which demonstrated that such relationship scenarios would be readily distinguished using this panel. We also observed more significant gaps between the full siblings and the unrelated individuals when the 133 iiSNPs were included into the case simulations. While for half sibling tests, significant LR distribution differences were detected between half sibling tests and the full sibling tests, and the overlap of the LRs indicated that a fair proportion of half sibling tests would be inconclusive in both the Yugu group and NMH population: unable to distinguish related and unrelated hypotheses. However, more conclusive results would be obtained when the set of 133 iiSNPs was incorporated in the half sibling tests. Using a stated threshold of 10,000 for the LRs, the exceedance probabilities were estimated to be 0.9980 (4A), 1.0000 (4B), 0.9930 (4C), and 1.000 (4D) for full sibling tests, while estimated to be 0.4580 (5A), 0.7740 (5B), 0.4380 (5C), and 0.7620 (5D) for half sibling tests in the Yugu and NMH populations, respectively.

#### Population Structure Generated by aiSNP Analysis

**FIGURE 6:**
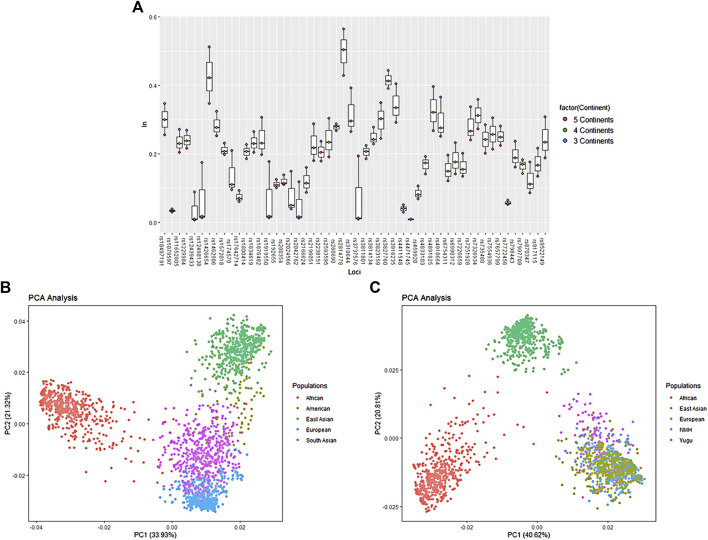
Ancestry inference efficacy evaluation among different intercontinental populations with 52 aiSNPs. **(A)** Ancestry inference informativeness (*I*_*n*_) of the 52 aiSNPs to distinguish populations from three, four, and five continents; **(B)** PCA scatter plot of 52 aiSNPs to distinguish five continental populations (excluding Yugu and NMH) at the individual level; **(C)** PCA scatter plot of the 52 aiSNPs to distinguish three continental populations (including Yugu and NMH) at the individual level.

The aiSNPs were further comprehensively analyzed to assess their capacities in assigning individuals to the corresponding biogeographic regions. The rs1871534 loci was excluded from further analysis for lack of genotype data of the reference populations. First calculated informativeness values (*I*_*n*_) of the 52 aiSNPs for distinguishing five, four and three continental populations, and then comparisons were made by presenting the overall results through the boxplot. The *I*_*n*_ values of the aiSNPs were been ranked in descending order and are listed in Supplementary Material. As shown in Figure 6A, the *I*_*n*_ values were labeled in red (five continents), green (four continents), and blue (three continents), and the topmost points were considered to be informative for ancestry inference in the corresponding continental populations. It was indicated that the set of aiSNPs was most valuable for differentiating three continental populations (Africa, Europe, and East Asia), followed by four continental populations (Africa, Europe, East Asia, and South Asia), and the least discrepancy power in five continental populations. We subsequently conducted the PCA based on the genotypic data of the overall samples from the five continental populations to display their clustering patterns. In Figure 6B, the first two factors accounted for 55.25% of the total variance. Factor 1 clearly separated the African populations from non-African populations, and factor 2 separated the East Asian populations from non-East Asian populations. The entire plot showed a certain tendency to distinguish African (red points), European (blue points), South Asian (purple points), and East Asian (green points) populations, but not informative to differentiate American population with the other continental populations. We also noted that the South Asian populations showed less clear separation among the other four continental populations. So, we further performed a refined PCA at three continental population level with individuals from the Yugu group and NMH population being recognized as targeted samples, and Figure 6C revealed that Africa, Europe, and East Asia populations were well differentiated with the first two principal components accounting for 61.43% of the total variance, and Yugu as well as NMH individuals clustered with most individuals of East Asian origin.

Based on the maximum-likelihood algorithm, the STRUCTURE analysis was used to further deeply characterize substructure patterns of the two studied populations and to infer their proportions of ancestral components at five continental population levels with the 52 aiSNPs. For a better understanding of the ability of the data to distinguish the most likely ancestry at the higher *K* values, the running times for each *K* was set to 15 replicates. The results for the specific structure run at each *K* value, *K* = 2–7, are shown in Supplementary Figure S6. At all *K* values, the populations exhibited quite homogeneous structure patterns in African, East Asian, South Asian, and European continental groups. The American populations displayed distinct genetic discrepancies with the increase of *K* values. By *K* = 4, the bar plot provided an obvious turning point to distinguish the ancestry compositions of the overall populations from five continents. The predominant ancestry of each population cluster was labeled in distinct colors when *K* = 5. Accordingly, the genetic ancestries of African, East Asian, European, and South Asian populations were represented by blue, light blue, green and pink lines, respectively. The principal ancestry components for the PEL and MXL population were represented by light green lines, which was slightly different from that of the other two American populations (CLM and PUR). Importantly, it was also discovered that a large fraction of East Asian genetic component was detected in the studied Yugu group and NMH population, suggesting that they were genetically close related to the East Asian populations. Besides, we also discovered that the Yugu group presented slight differences in genetic ancestry compositions with NMH and the other East Asian populations and when *K* = 3–5, the differences were more obvious from the bar plots. The results for the specific structure runs with the highest likelihood at *K* = 3 (the bar plot and the line chart) along with the clustering patterns (the triangle plot) are shown in Supplementary Figure S7. So, we further ran the STRUCTURE analysis to infer ancestry component proportions for the Yugu and NMH populations at the three continental population levels. At the best-fit model of *K* = 3, highly specific genetic components were observed in populations from the same continent. Accordingly, the genetic ancestries of the African, European, and East Asian populations are represented by blue, red, and purple lines, respectively. The East Asian ancestry components for the Yugu group and NMH population were estimated to be 92% and 99%, respectively. The triangle plot also indicated that the Yugu individuals (labeled in green dots) and NMH individuals (labeled in purple dots) were clustered with the other East Asian individuals (labeled in yellow dots) and were positioned at the vertex of the triangle.

The performances of the piSNPs to predict the physical characterizations of the Yugu and NMH individuals are detailed in the Supplementary Material. As was indicated, the recruited individuals revealed 100% of brown eyes and black hair in both the Yugu and NMH population. The dry earwax type was overwhelmingly detected in the Yugu (78.18%) and NMH (94.89%) individuals, which indicated a prevalence of dry earwax in the NMH population based on the present finding. The detection of lactose intolerance manifested that the individuals from Yugu and NMH showed deficiencies in metabolizing lactose. Muscle performance was rarely focused in forensic science. But the predictions indicated that the majority of Yugu (84.85%) and NMH (84.68%) individuals exhibited sprinter potencies, which indicated well muscle performances among the two analyzed populations. The results of alcohol flush reaction inference gave insights into the fact that most individuals from the Yugu group (81.21%) and NMH population (70.57%) showed little or no flush reactions after intake of alcohol.

### Mitochondrial DNA–Based Analysis

#### Sequencing Depth of Mitochondrial DNA Hypervariable Region

The mtDNA hypervariable regions were divided into seven fragments (MT1: 22-274; MT2: 154-430; MT3: 363-542; MT4: 488-639; MT5: 15933-16162; MT6: 16114-16343; and MT7: 16199-16456) to be amplified by the MGI system. The total read depths for the seven amplicons ranged from 979,070× at MT3 to 12,255,484× at MT7, with the average read depth spanning from 1,966× to 24,609×. The sequence performance revealed that the MGI sequencing platform could perform well in the sequencing of the mitogenome hypervariable regions.

### Haplogroup Assignment of Yugu and NMH Populations

All 498 complete mitotypes were classified into 46 specific sub-haplogroups in the Yugu and NMH populations according to the PhyloTree build 17. Detailed haplogroup and sub-haplogroup affiliations of the analyzed samples are listed in the Supplementary Material and further visualized by different pie charts. Among the assigned haplogroups in the Yugu group (Supplementary Figure S8A), macro-haplogroup M was the most frequent haplogroup (67%), followed by haplogroup R (23%) and N (10%). In detail, D4 (26%) (namely, D4a, D4b, D4c, D4g, D4h, D4j, and D4m) exhibited the highest frequency in the M macro-haplogroup, followed by D5 (8%), M9 (8%), and other sub-haplogroups. We used M* to represent the other sub-haplogroups of the M haplogroup with lower frequencies in the Yugu group. The other haplogroups C4, M8, G3, G2, E1, and Z were also observed with proportions of 6%, 4%, 4%, 3%, 2%, and 2%. The R macro-haplogroup also comprised plenty of sub-clades, namely, haplogroups F, B4, B5, H, R, and T in this study. The F haplogroup was most prevalently detected with a proportion of 10% in the R macro-haplogroup, followed by B4 (4%) and B5 (4%). The N macro-haplogroup was less frequently observed in the Yugu group, which comprised the sub-haplogroups of A (9%) and N9 (2%).

In the NMH population (Supplementary Figure S8B), the M macro-haplogroup was also most frequently observed with a proportion of 53% of the total samples, followed by R (30%) and N (17%). But the haplogroup composition of M macro-haplogroup in the NMH population was considerably different from the sub-clades of M in the Yugu group. As expected, the haplogroup D4 (namely, D4a, D4b, D4c, D4e, D4g, D4h, D4i, D4j, D4o, D4q, and D4t) was the most prevalently detected sub-haplogroup among M (14%), followed by M7 (6%), M*(6%), G (6%), D5 (5%), M9 (5%), C4 (6%), C5 (2%), M8 (2%), E1 (2%), Z (2%), and C (1%). The M* haplogroup was composed of a collection of M sub-haplogroups with low frequencies, we therefore did not separately discuss M* here. In general, the D haplogroup took up the largest proportion of the M macro-haplogroup. Among the R macro-haplogroup, the F1 lineage (10%) was the most prevalently observed sub-clade, followed by B4 (8%), F* (4%), R (3%), B5 (2%), H (1%), and U (1%). The N macro-haplogroup was composed of A (10%), N9 (6%), and Y (1%) haplogroups. According to previous studies, the East Asian–specific lineages were reported to contain haplogroups C, D, G, Z, E, M9a, M7, M13, A, B, F, R9c, Y, and N9a. So, the prevalent haplogroups detected in Yugu and NMH were basically identical to the previously observed haplogroups (Umetsu et al., 2005; Li et al., 2019).

### Statistical Indexes Estimation of Mitochondrial DNA Hypervariable Region in Yugu and NMH Populations

As indicated by DnaSP (Table 2), a total of 110 (Yugu) and 272 (NMH) haplotypes were estimated from 498 mtDNA sequences. The haplogroup diversities were 0.9932 in the Yugu group and 0.9979 in the NMH population, respectively. The other statistical indexes including the number of segregating sites (s = 115 for Yugu and s = 166 for NMH) and the average number of pairwise nucleotide differences (*k* = 7.976 for Yugu and *k* = 8.414 for NMH) were also estimated to be relatively informative. Moreover, the neutrality tests presented significantly negative values, encompassing Tajima’s D (−1.9817, *p* < 0.05 for Yugu; −2.1137, *p* < 0.01 for NMH) and Fu and Li’s F tests (−2.7671, *p* < 0.05 for Yugu; −3.1762, *p* < 0.02 for NMH). Mismatch distributions were also implemented to infer the histories of the Yugu and NMH populations with a unimodal pattern, and the model test statistics, including SSD and HRI, were performed to evaluate whether the mismatch distributions were significantly divergent from the expected expansion model. Based on the estimated SSD and HRI, no significant deviations from the model expectation of demographic expansion were detected in the Yugu group (0.00074, *p* = 0.44; 0.00622, *p* = 0.56) and NMH population (0.00069, *p* = 0.1; 0.00627, *p* = 0.38).

**TABLE 2:** Diversity indexes and neutrality tests for the studied Yugu ethnic group and NMH population.

Populations	N	H	Hd	Gene Diversity			Neutrality Test		Mismatch Distribution	
				S	k	Pi	Tajima’s D *(p)*	Fu and Li’s F *(p)*	SSD *(p)*	HRI *(p)*
Yugu	165	110	0.9932	115	7.976	0.0107	−1.9817 (<0.05)	−2.7671 (<0.05)	0.00074 (0.44)	0.00622 (0.56)
NMH	333	272	0.9979	166	8.414	0.0115	−2.1137 (<0.01)	−3.1762 (<0.02)	0.00069 (0.1)	0.00627 (0.38)

Note: N, sample size; H, haplotype; Hd, haplotype diversity; S, number of segregating sites; k, average number of nucleotide differences; Pi, nucleotide diversity; SSD, sum of squares of deviations; HRI, Harpending’s raggedness index.

### Genetic Divergence Among the Analyzed Populations and the Reference Populations

**FIGURE 7:**
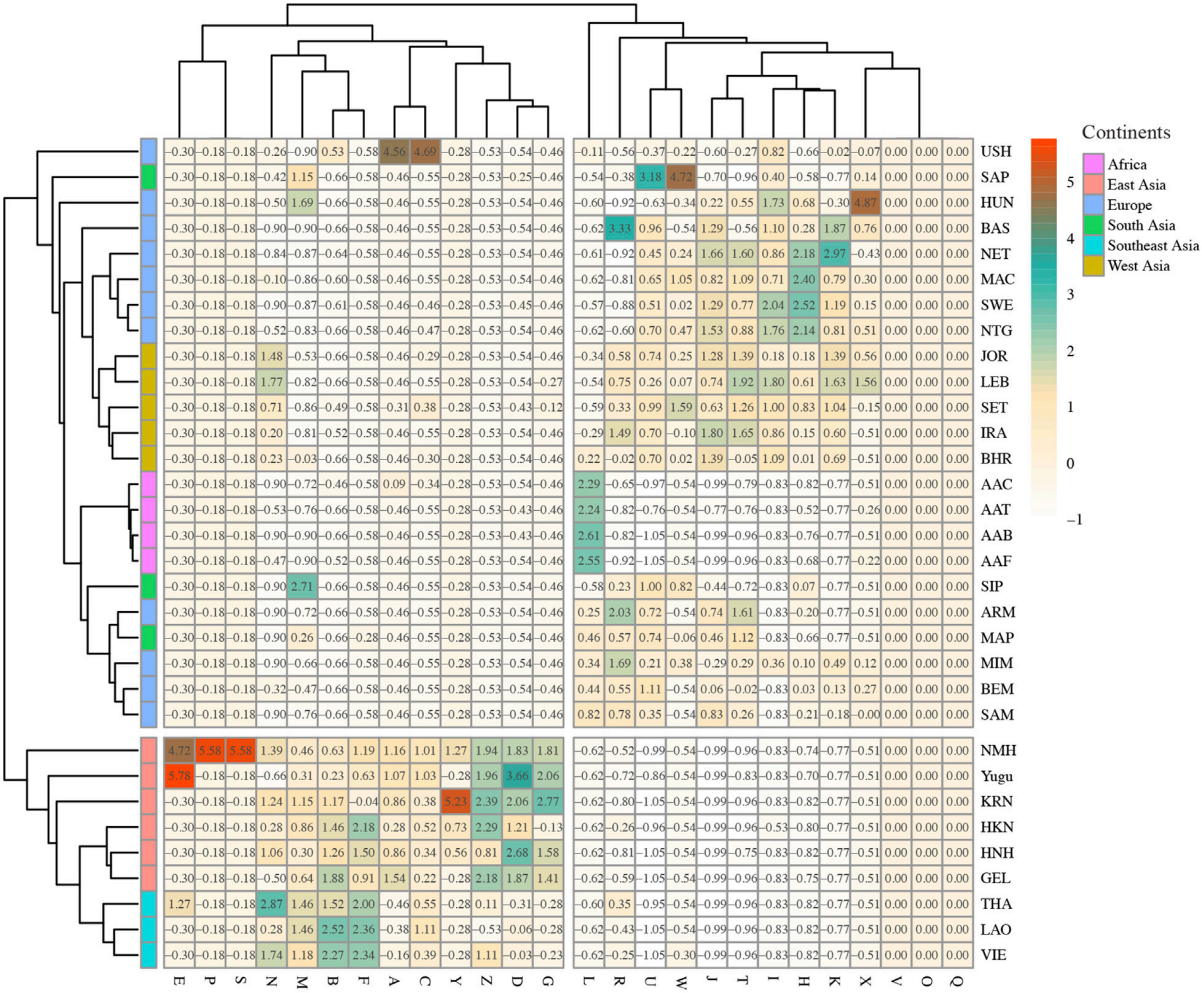
MtDNA haplogroup frequencies of the two studied populations and the reference populations. The frequencies of haplogroups are represented by the color gradient. The color gradient for mtDNA haplogroup frequencies ranges from white to red, corresponding to the mtDNA haplogroup frequencies from low to high.

The reference populations were integrated from previous studies to investigate the genetic relationships of the studied populations and the comparison populations from the matrilineal phylogenetic perspective. Detailed information of the reference populations is listed in the Supplementary Material. MtDNA haplogroup distributions of the recruited populations were visualized through the heat map. As shown in Figure 7, the gradient of the haplogroup frequencies ranged from white (corresponding to low haplogroup frequencies) to red (corresponding to high haplogroup frequencies). We noticed that the differentiated frequencies of these dominant haplogroups were detected in different continental populations. But in general, populations from the same continent shared basically analogous frequency distributions and clustered into the same subbranch of the polygenetic tree. The Southeast Asian populations assembled with the East Asian populations, which could be explained by their close biogeographic distances and possible gene interchanges. The mtDNA haplogroup frequencies in Yugu and NMH populations were most similar with those of East Asian reference populations.

Based on haplogroup frequencies, the PCA was performed to explore the genetic relationships between the studied populations and the comparison populations (Supplementary Figure S9). From PC1 and PC2, five distinct clusters could be observed, these were the African cluster, European cluster, South Asian cluster, West Asian cluster, and the East Asian–Southeast Asian cluster. The PCA plot displayed distinct divisions between the African populations and non-African populations, as well as the East Asian and Southeast Asian populations and the other referenced populations. Of note is that the East Asian and Southeast Asian populations clustered tightly in the PC plot, which was consistent with their haplogroup frequency distributions. Besides, the West Asian populations and South Asian populations basically clustered on the left side of the map, with the European populations dispersedly distributed in the PCA plot. As is indicated by Supplementary Figures S9A,B, the first two PCs almost took 89% of the total variance. From Supplementary Figures S9C,D, it was suggested that the haplogroup frequency differences of B, D, F, H, L, and M could explain the scattered distribution of the populations from PC1 level, especially haplogroup L. And haplogroup frequency differences of B, D, F, H, M, R, T, and U could explain the scattered distribution of the populations from PC2 level.

Pairwise *F*_*ST*_ values were also estimated based on haplogroup frequencies of the overall populations. Then we visualized the *F*_*ST*_ values between the Yugu and comparison populations as well as NMH and the reference populations through circle bar plots. As shown in Supplementary Figure S10A, it was indicated that the populations from the same continent shared basically analogous *F*_*ST*_ values, which was measured by the height of the bar plot. The African populations exhibited the largest pairwise *F*_*ST*_ with Yugu (labeled in red), followed by the European populations (in green), South Asian populations (in cyan), West Asian populations (in purple), Southeast Asian populations (in blue), and East Asian populations (in brown). It was also indicated that the HNH population presented the most marked genetic affinities with the Yugu group, followed by the NMH population. For the NMH population (Supplementary Figure S10B), basically identical pairwise *F*_*ST*_ distributions were discovered between the NMH and reference populations. That is, the East Asian populations presented the most remarkable genetic affinities with the NMH population (an averaged *F*_*ST*_ value of 0.0052), followed by the Southeast Asian populations. The West Asian and South Asian populations showed the intermediate genetic relatedness with the NMH population, and the African populations displayed the most genetic divergences with the NMH population (an averaged *F*_*ST*_ value of 0.4005). The pairwise *F*_*ST*_ results also indicated that the East Asian populations, especially the Han population from the northern region of China implied closer genetic affinities with the Yugu and NMH populations. Detailed pairwise *F*_*ST*_ values are presented in the Supplementary Material.

### Haplotype Sharing Between the Studied Populations and the Reference Populations

**FIGURE 8:**
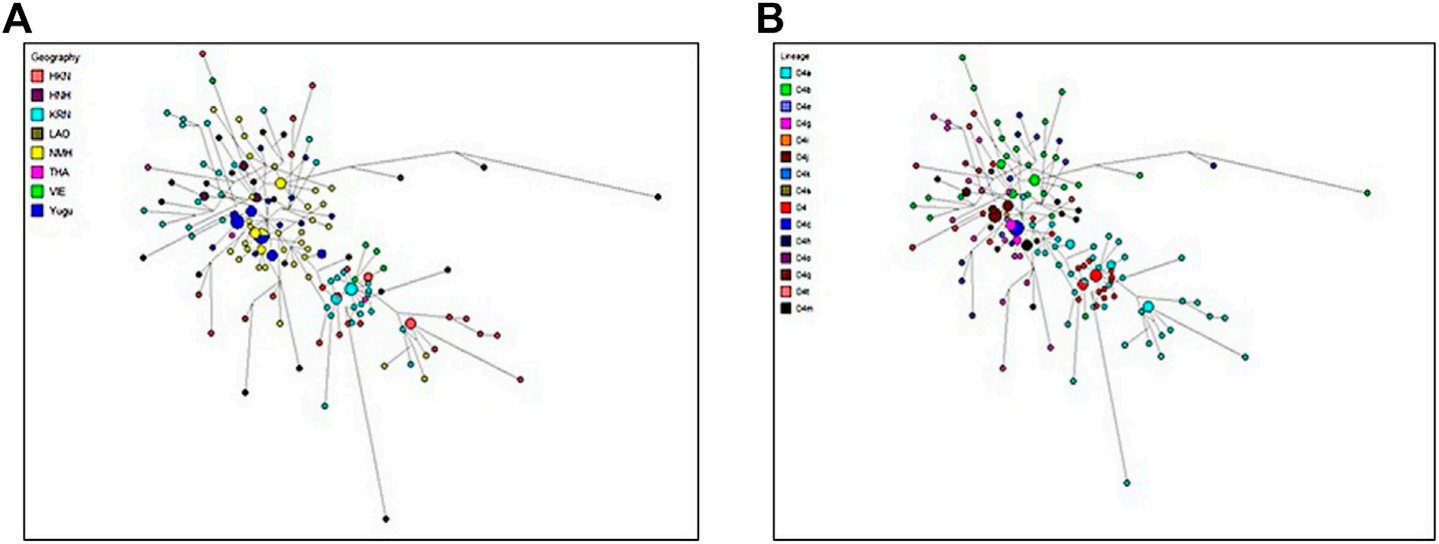
The median networks of mitochondrial D4 haplogroup. **(A)** D4 haplogroup colored by the geographic origins of the populations; **(B)** D4 haplogroup colored by lineages of the populations. Different colors represent different continental origins or different haplogroup lineages, and the size of the circle is proportional to the number of individuals.

From the abovementioned genetic analysis, the results suggested that the Yugu and NMH populations revealed more genetic relatedness with the East Asian populations (KRN, HKN, HNH, and GEL), followed by the Southeast Asian populations (THA, VIE, and LAO). But to comprehensively explore the genetic sharing between the two studied populations and referenced populations from the East Asia and Southeast Asia, we conducted haplotypic comparisons of the 2051 samples and then performed the median-joining network analysis with the most prevalent haplogroups from the perspectives of the geographic regions (Figure 8A) and haplogroup lineages (Figure 8B). The analysis also indicated that the predominant haplogroups of the East Asian and Southeast Asian populations were assigned to Asia-specific haplogroups, and given the limited sizes and the widespread haplotype distributions of the recruited populations, all the present haplotypes of the two populations were considered in this analysis. Besides, one mutation difference of the haplotypes between the studied individuals and the referenced individuals was simply counted based on network results. As previously discussed in the pie chart analysis, 165 Yugu individuals fell within 20 different haplogroups, namely, haplotypes belonging to macro-haplogroup M, macro-haplogroup N and macro-haplogroup R. For the geographical assignment of D4 haplogroup (Figure 8A), it was suggested that Yugu individuals were predominately clustered with individuals from the NMH and HNH populations. To be specific, most Yugu individuals displayed the same haplotypes with NMH individuals or differed from the NMH and HNH individuals by only one mutation, reflecting close genetic relatedness between the Yugu and Han individuals of different regions. In addition, we found that two NMH individuals and one NMH individual shared one mutation with KRN population and HKN population, respectively. From the haplogroup lineage aspect, the Yugu and NMH individuals tended to share the same haplotypes or differ by one mutation on the D4 haplogroup and four sub-haplogroups D4a, D4b, D4g, and D4j, as indicated by the small circles labeled in various colors in the plot (Figure 8B).

### Demographic History Analyses of Yugu and NMH Populations

**FIGURE 9:**
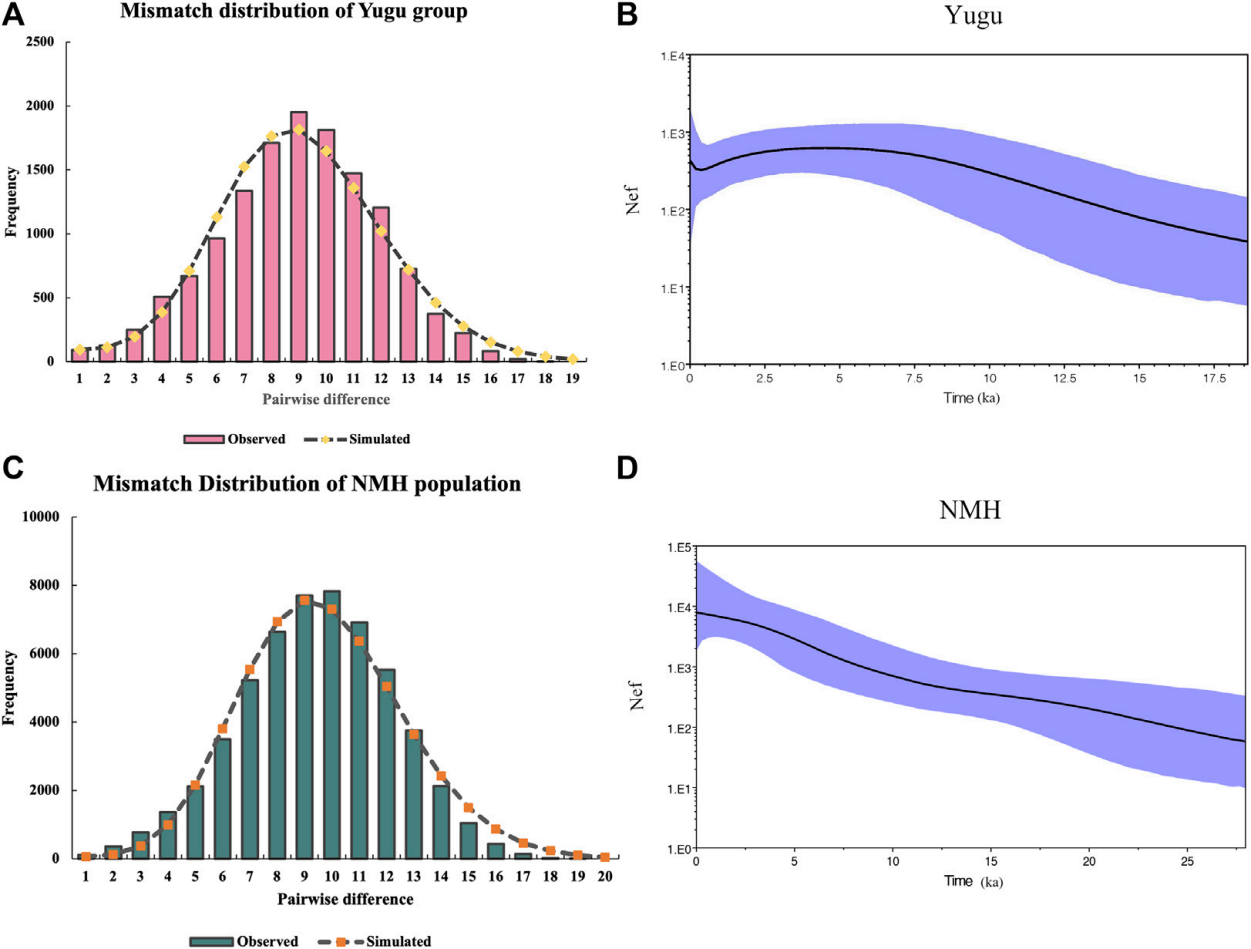
Mismatch distribution and Bayesian skyline plot (BSP) analyses of the Yugu ethnic group **(A,B)** and NMH population **(C,D)**. The *X*-axis of mismatch distributions indicate pairwise differences between the observed and simulated sequences. The *Y*-axis of BSP plots represent the assumed effective population size on a log scale. The blue lines in bold represent the median population.

As depicted above, a series of analyses have implied that the Yugu group and NMH population shared closer genetic relatedness with East Asian and Southeast Asian populations, and to be more exact, the Yugu group showed more genetic similarities with NMH in the present study. Then, we performed BSP analyses based on the sequences of mtDNA hypervariable regions to explore the demographic histories of the Yugu and NMH populations. As indicated in Figure 9A and Figure 9C, mismatch distributions of Yugu and NMH populations indicated the occurrence of population expansion through time. Subsequently, BSP representing the assumed effective population size of the Yugu group (Figure 9B) and NMH population (Figure 9D) were generated. It was noted that the population increase for the Yugu group occurred around 12.03 ka, a time period belonging to the Late Pleistocene. In the NMH population, the population increase was displayed since 11.29 ka, a time period at early Holocene.

## Discussion

The advanced progress in MPS technology tremendously reduces experimental costs and enables the combination of much-expanded number of genetic markers with the aim to increase evidence weight in forensic casework. The newly developed MGI identification system possesses the ability to simultaneously detect six kinds of nuclear genetic markers and mtDNA variations, which has been proved to be a very promising tool for forensic DNA analysis. Compared with the commercially available MiSeq™ FGx System, the MGI system comprehensively embraced a wide variety of DNA markers to fulfill the demands of forensic practice.

For the first time, the MGI identification system was comprehensively assessed for forensic purpose and population genetic analysis in 165 Yugu and 333 NMH individuals. In terms of the frequently used descriptive parameters for forensic purpose, like Hobs, heterozygosity and PIC, previous reports have declared that the genetic marker with heterozygosity value over 0.5 and PIC over 0.5 could be treated as highly informative for genetic diversity investigations (Zeng et al., 2016; Yousefi et al., 2018; Jin et al., 2020). The PM is defined as the probability of a match between two randomly selected individuals (Balding and Nichols, 1994). The smaller the PM is, the higher the evidential value that the targeted genetic marker can provide. The PE is a necessary parameter in the paternity test scenario that can estimate the probability of excluding a random parent pair related to the tested child (Rapone et al., 2016). A higher PE demonstrates a more robust power for forensic parentage testing. In this study, the highly polymorphic genetic markers are carefully selected and incorporated into the panel. The estimation of the statistical parameters for A-STRs, X-STRs, Y-STRs, and iiSNPs indicate that the MGI identification system could yield more genetic variations and provide adequate discrimination power for individual identification and parentage testing in forensic practice. The advantage of allele increment for the A-STR loci is discussed here. Compared with traditional length-based polymorphism detected by the CE method, total alleles discrepantly increased due to the SNP variations in the motifs at different STR loci in Yugu and NMH populations. Specifically, the top five loci presenting the most significant contribution to sequence polymorphism increment in their repeat regions were D8S1179, D7S3048, D21S11, D11S2368, and D8S1132 in the Yugu group, while D13S317, D8S1132, D11S4463, D7S3048, and D2S1338 in the NMH population. Additional alleles of the D7S3048 locus were observed to considerably increased in both the Yugu and NMH populations, but the D13S317 locus displayed the most allele increment in the NMH population, while displaying relatively less allele increment in the Yugu group, which indicated that optimal selection of the STR loci in a MPS-based panel should be considered in further work. Besides, the allele gain of the D7S3048 locus was also consistent with a previous study (Liu et al., 2020). Nonetheless, the undetectable allele gains were observed at several loci in the Yugu and NMH populations, which should be further verified in other populations. The Y-STRs and X-STRs were also demonstrated to be highly polymorphic and efficient enough for patrilineal- and matrilineal-related complex kinship determinations.

When analyzing the 145 iiSNPs, 12 iiSNPs of which were removed from further analyses for significant deviations from the HWE in both the Yugu or NMH populations. We subsequently testified that the highly homologous sequences in the flanking regions of the 12 iiSNPs, which caused nonspecific amplification during PCR. The forensic parameters for the 133 iiSNPs, including Hobs, PD, PM, PIC, MP, and GD values indicated less discrimination power than that of the 54 A-STRs. But the distribution of CPM demonstrated that less than 40 iiSNPs were capable of meeting the theoretical probability of individual identification for forensic science. To our knowledge, the CPM of the 133 iiSNPs was lower than that of the 94 iiSNPs in the ForenSeq™ DNA signature Prep Kit (Delest et al., 2020). With 54 A-STR and 133 iiSNP loci simultaneously detected, cumulative discrimination power of the novel assay was significantly elevated, which was comparable to efficiencies of 74–90 A-STRs as previously described (Li et al., 2021) and further proved in this study. We also put forward that another advantage of the MGI identification system was the relatively small amplicons in the panel, to show a better performance in challenging DNA profiling, such as degraded DNA samples.

Genetic structures of the two studied populations were systematically explored in this study. From the patrilineal landscape of Y-STR analysis, the Yugu and NMH populations were discovered to share genetic affinities with the East Asian populations based on pairwise *F_ST_* values, closer clustering patterns of MDS, and phylogenetic relationships. Deeper investigation into the East Asian populations revealed that the Yugu and NMH populations exhibited more genetic similarities with most Han populations from different geographic locations, especially northern Han populations, such as LNH and SDH. The efficiencies of the aiSNPs in assigning unknown individuals to the corresponding populations of African, East Asian, South Asian, and European origins were also proved here. But a precise prediction would be acquired when the ancestry prediction of unknown individuals was confined to three continental populations (African, European, and East Asian).

For iiSNP analysis, relationship test simulations, which included full sibling and half sibling determination cases, were also performed to test their kinship prediction efficiencies. The results illustrated that the inclusion of the 133 iiSNPs for the simulations gave much higher likelihood ratios in the analyses of full sibling tests in the Yugu and NMH populations but was slightly higher for the half sibling tests. Despite the lack of power to adequately distinguish the relatively challenging half sibling relationships, the recruited iiSNPs evidently showed capacity in separating the half siblings and unrelated individual pairs. For this reason, it was indicated that the MGI identification system was informative for complex kinship identifications to some extent. In addition, kinship determination was also informative for establishing links between the unknown individuals and their surviving relatives in criminal cases, which was proved by the Gold State Killer case (Hazel et al., 2018). Therefore, incorporating a large number of highly polymorphic SNPs and STRs in a MPS-based assay represents a major step forward in forensic complex kinship determination and genealogy study. Phenotype-related SNPs possess tremendous power in providing informative clues to catch unknown individuals, but owing to inherent traits of most Chinese populations, frequently studied piSNPs (like eye color and hair color piSNPs) could not reveal their potential discrimination capacities, which was confirmed in the present study. But the newly incorporated four characteristics including earwax type, lactose intolerance, muscle performance, and alcohol flush reaction presented their potential discrimination power in the Yugu and NMH populations. Further work is encouraged in more populations to testify the efficiencies of piSNPs to predict individual physical characteristics.

MtDNA landscapes of the Yugu and NMH populations were described in this study by analyzing the mtDNA hypervariable regions in 165 Yugu and 333 NMH individuals. First, haplogroup allocations of the recruited participants indicated the prevalence of East Asian–specific haplogroups in the two analyzed populations. The most typical D4 haplogroup was previously reported in high frequencies in Northeast Asian populations and Han populations from North and Northeast China (Umetsu et al., 2005; Li et al., 2019), which was proved in this study. Second, by integrating genotype data of the reference populations, it was indicated that the studied Yugu and NMH populations exhibited analogous haplogroup distributions and minor *F*_*ST*_ values with the East Asian and Southeast Asian populations. Especially, the Han population from the northern region of China (like HNH) revealed the least pairwise *F*_*ST*_ values with Yugu and NMH populations, hinting at the close genetic relationships among the abovementioned populations. Of note is that the Yugu ethnic group and NMH population presented closer genetic affinities with the KRN population based on mtDNA variations, which could probably be explained by their close geographic locations. Furthermore, the population structures and mtDNA diversities of the network analyses also supported the close genetic relatedness between Yugu, NMH, and northern Han population. But we must admit that current notions are limited and could be further refined by recruited more reference populations into the matrilineal analysis. Third, by synthesizing population genetic analysis and demographic history analyses, the results implied that population expansions may have occurred in these two populations during Late Pleistocene (Yugu) and early Holocene (NMH). But owing to the discrepant substitution rates of a single nucleotide in the complete mitogenome and the control region, the presently estimated time nodes were discrepant with previous studies (Li et al., 2019; Chen et al., 2020b).

The Yugu group primarily lived in the Gansu province of northwest China. According to the historical documents, the Yugu group was recorded to originate from the Huangtou Huihu tribe which could be traced back to the Tang dynasty. The development and formation of the Yugu group was a complex process. The Yugu people have undergone gene communications with populations of different geographic locations in their migration history, like the Han population in the South, minorities in Xinjiang, and populations in the Hexi Corridor (Yang and Yu-June, 2016). Beyond that historical factors during the medieval Chinese dynasties, such as the Tang, Song, and Yuan Dynasties, largely promoted population interflows (Li, 2009; Song, 2019), especially, the territorial expansion of the Mongol Empire facilitated interactions between the Mongols and populations of their controlled regions, thus leaving notable influence on the genetic background of the old Yugu people (Zhao and Lin, 2020). Furthermore, previous studies also claimed that the Tibetan culture and the neighboring populations of the Yugu group (like Tu nationality) have also contributed to the formation of the Yugu group (Yang and Yu-June, 2016). Research on the genetic background of the Yugu group is surely a benefit for revealing its origin and formation process. From the genetic perspective, the genetic distributions of A-STRs (Chen et al., 2006), X-STRs (Chen et al., 2008), InDels (Yang et al., 2019), and HLA alleles (Yao et al., 2012) have been explored in the Yugu group, and valuable genetic information was accumulated. The close relationships among the Tibetan, Mongolian, and Yugu groups and the Han population were revealed based on the genetic distributions of the X-STRs (Chen et al., 2008). While the HLA allele and haplotype distributions yielded that the Yugu group shared close genetic affinities with the Tu and Hui groups and the Han population (Yao et al., 2012).

To sum up, the precise genetic background of the Yugu group still remains unclear even to this day. In the present study, genetic insights from the perspectives of autosomal DNA, Y-chromosomal DNA, and mtDNA variations have yielded that the Yugu group was genetically close related with the Han population of the northern region, as well as with the NMH population. As is indicated, Yugu and NMH individuals were sampled from the Gansu province and Inner Mongolia Autonomous Region, respectively. These two locations were regarded as the genetic treasures of the Chinese nation for being settlements of many ethnic minorities. Besides, the two regions are geographically neighbors to each other from Northern China. It is reasonably speculated that geographic locations, society, and economic factors could play certain roles in population interchanges of the two locations. However, since we only incorporated a limited number of ethnic groups in this study, more relevant groups (like Mongolian, Tibetan, Hui, and Tu) should be incorporated to gain a refined genetic composition landscape of the Yugu group.

## Data Availability

The raw genotype data used and analyzed during the current study are available from the corresponding author on reasonable request.
