# Supplementary material for: Forensic Feature Exploration and Comprehensive Genetic Insights Into Yugu Ethnic Minority and Northern Han Population via a Novel NGS-Based Marker Set
Source: Front Genet. 2022 Apr 27;13:816737. doi: 10.3389/fgene.2022.816737 (PMC9121381; doi:10.3389/fgene.2022.816737)
Supplement: Supplementary file 2 [file Image1.pdf]

Supplementary Figures

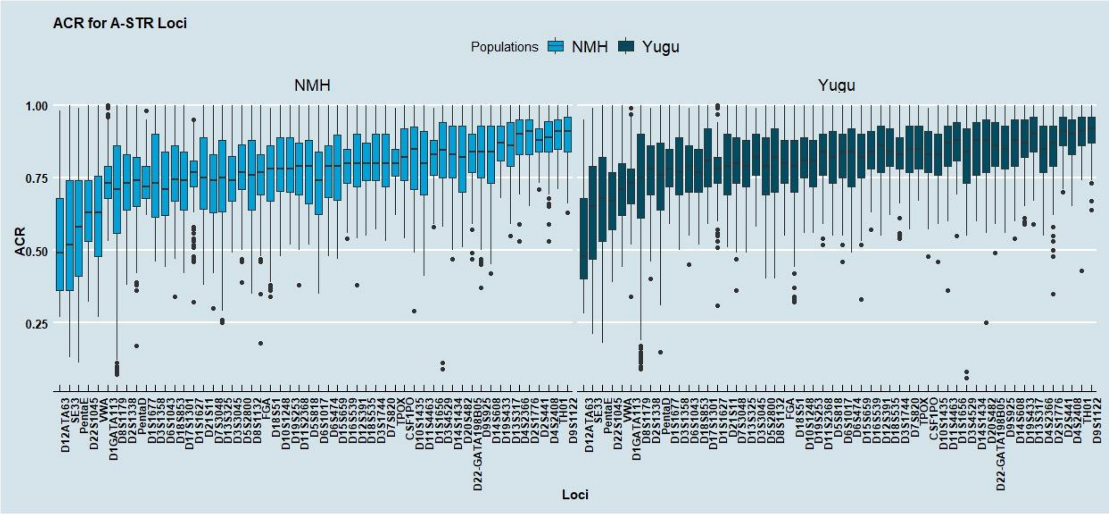

Supplementary Figure S1: Allele Coverage Ratio (ACR) of the 54 A-STRs in Yugu and NMH population. The A-STRs are arranged by averaged ACR in ascending order.

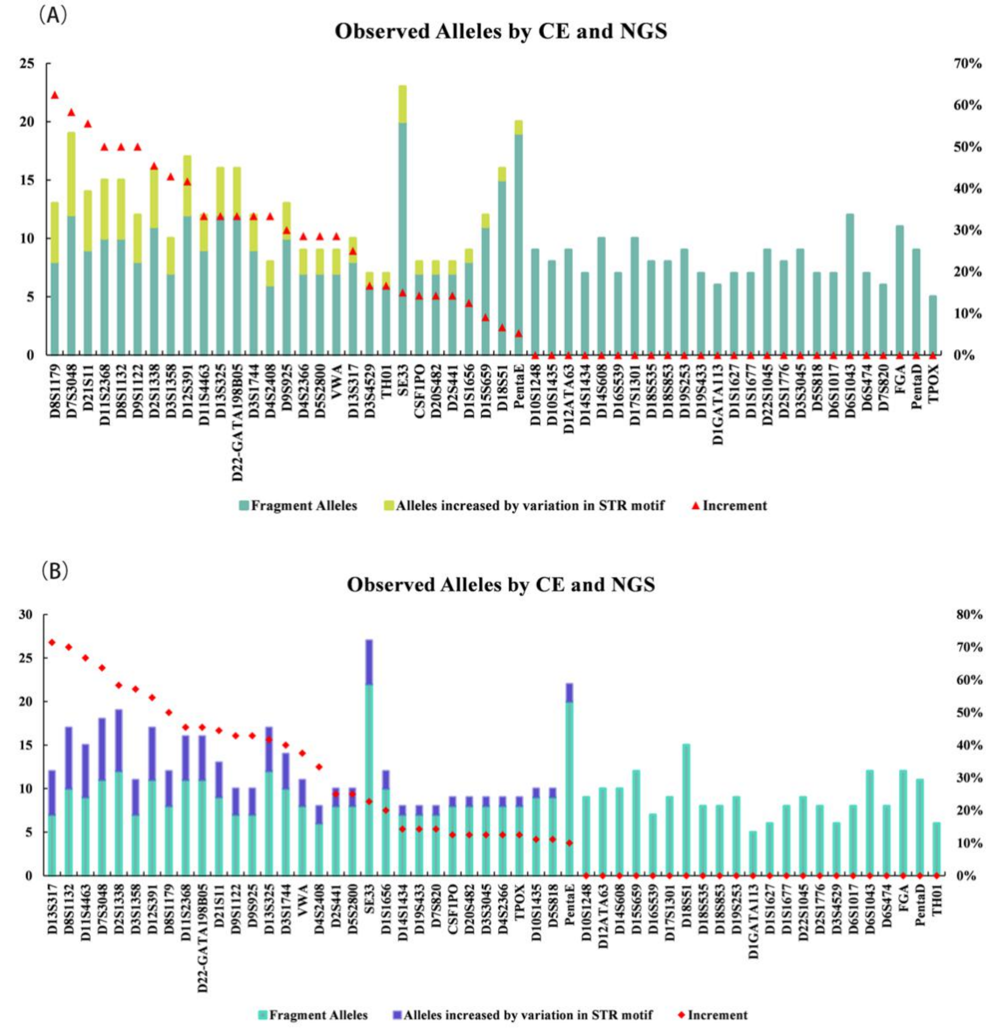

Supplementary Figure S2: (A) Comparison of observed alleles by CE and NGS in Yugu ethnic minority. The green bar represents length-based alleles by CE and the brown bar represented additional sequenced-based alleles by NGS. The red triangle indicates increment of additional alleles observed by NGS; (B) Comparison of observed alleles by CE and NGS in NMH population. The green bar represents length-based alleles by CE and the brown bar represented additional sequenced-based alleles by NGS. The red triangle indicates increment of additional alleles observed by NGS.

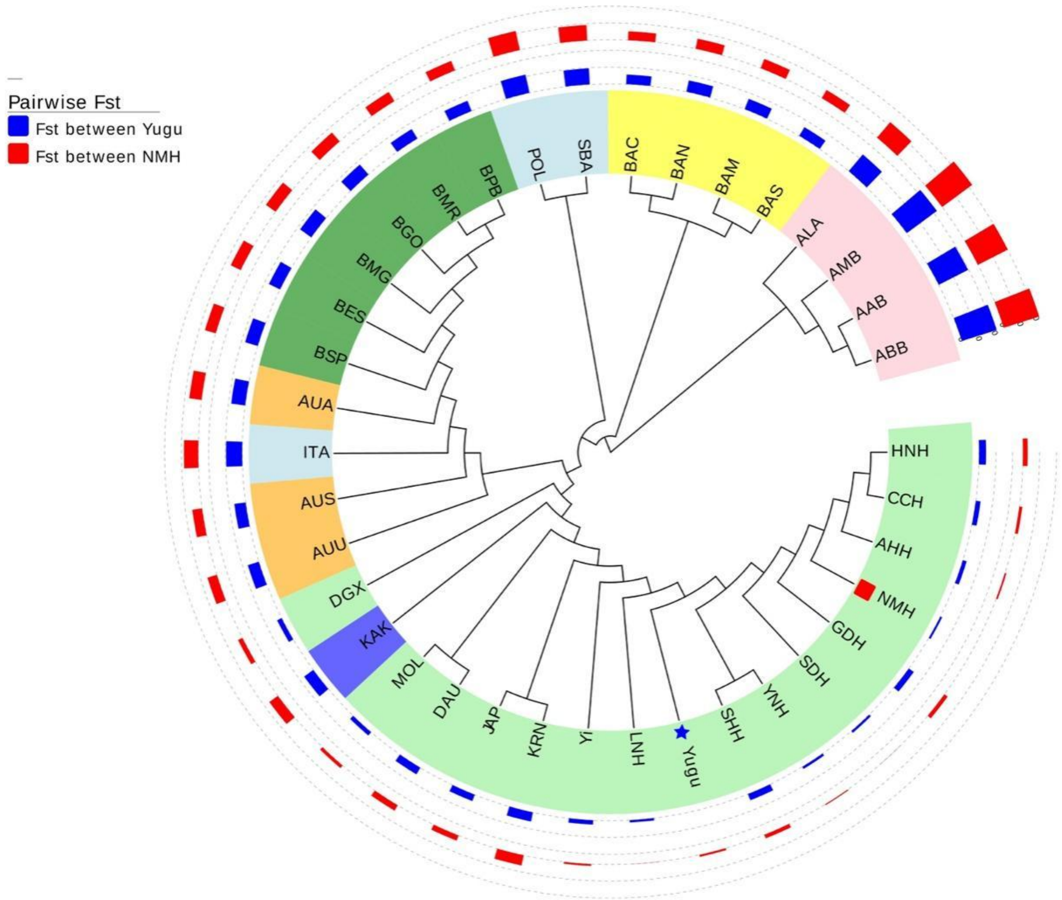

Supplementary Figure S3: Phylogenetic tree and pairwise F_{ST} values among the two studied populations and the reference populations based on the Y-STR genotype data. The bars outside of the circle phylogenetic tree represent the pairwise F_{ST} values between the Yugur (blue bar) ethnic minority and the referenced populations, as well as the Inner Mongolian Han (red bar) population and the referenced populations, respectively.

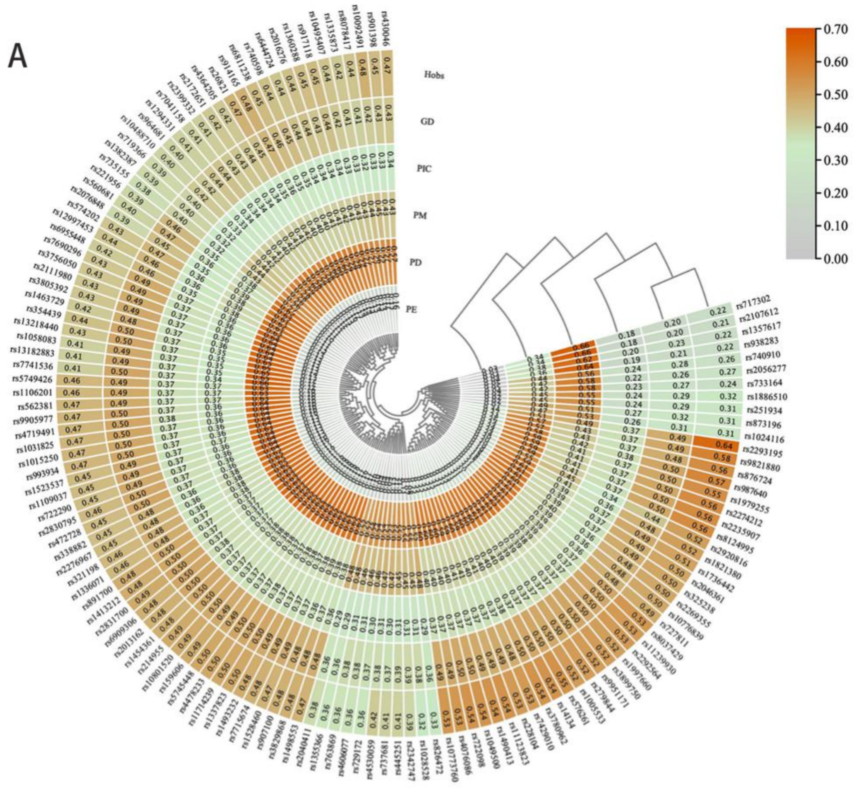

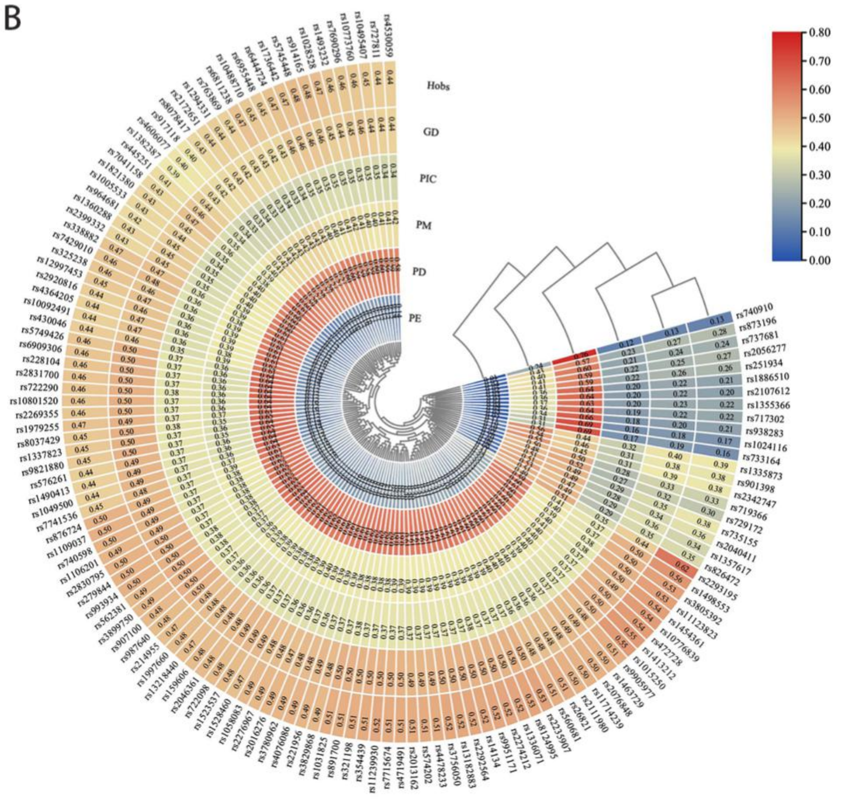

Supplementary Figure S4: (A) Forensic descriptive parameters of iiSNPs in Yugu ethnic group. The color gradient for the parameters ranges from gray to red, corresponding to the parameters from low to high; (B) Forensic descriptive parameters of iiSNPs in NMH population. The color gradient for the parameters ranges from blue to red, corresponding to

the parameters from low to high.

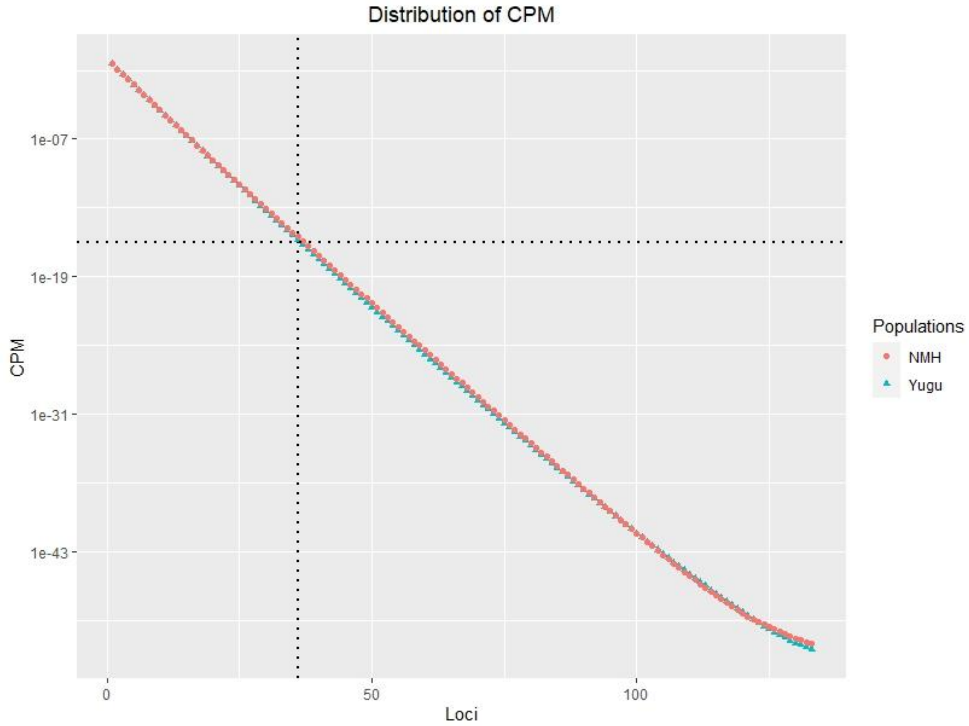

Supplementary Figure S5: Change of cumulative match probability (CMP) with the number of iiSNPs in Yugu ethnic group and NMH population. The iiSNPs are sorted by their discrimination power in descending order.

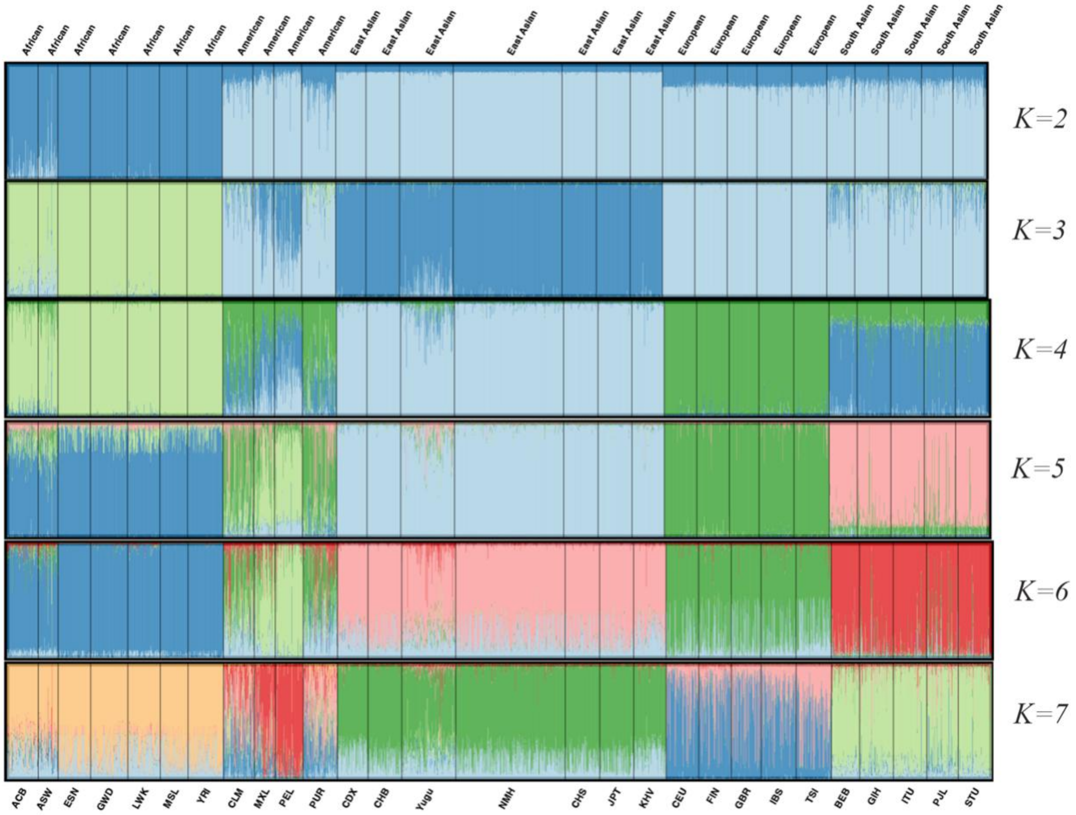

Supplementary Figure S6: Population genetic architecture prediction at five continental populations level based on 52 aiSNPs with STRUCTURE software. The pre-assumed ancestries (*K*) are defined from 2~7.

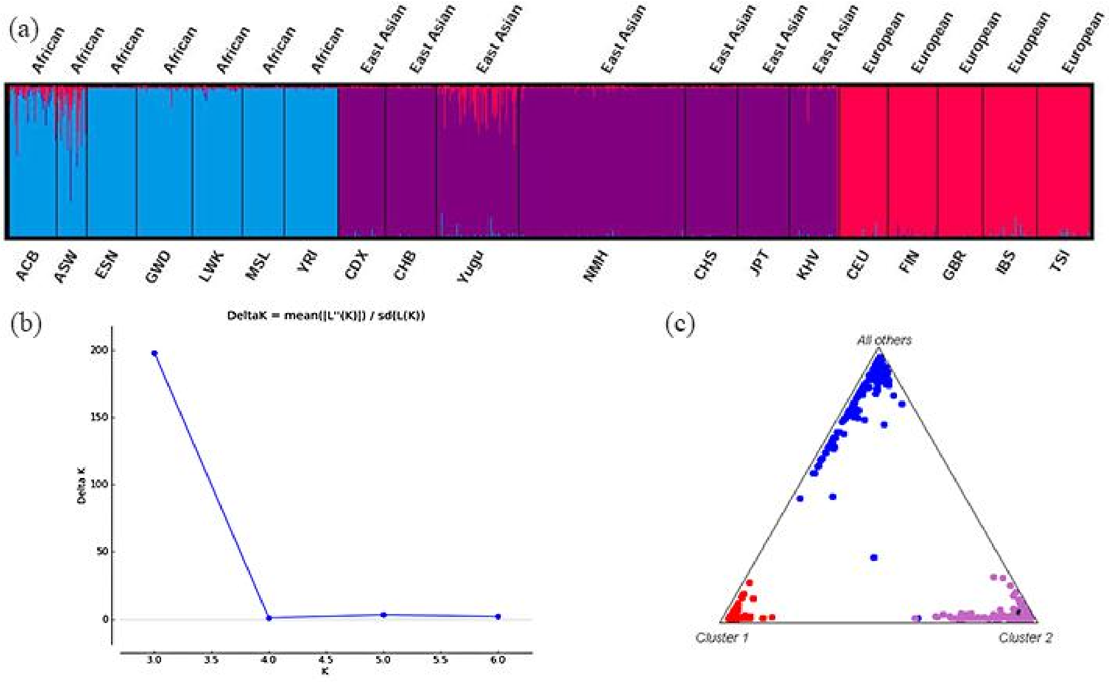

Supplementary Figure S7: Results for the specific structure runs with the highest likelihood at *K* = 3 (the bar plot and the line chart) along with the clustering patterns (the triangle plot) based on STRUCTURE analysis. (A) Population genetic architecture prediction at 3 continental populations level based on 52 aiSNPs; (B) Optimal *K* determined based on 5 continental populations; (C) Triangle plot showing clustering pattern of the analyzed populations and referenced populations from three continents (Africa, Europe and East Asia).

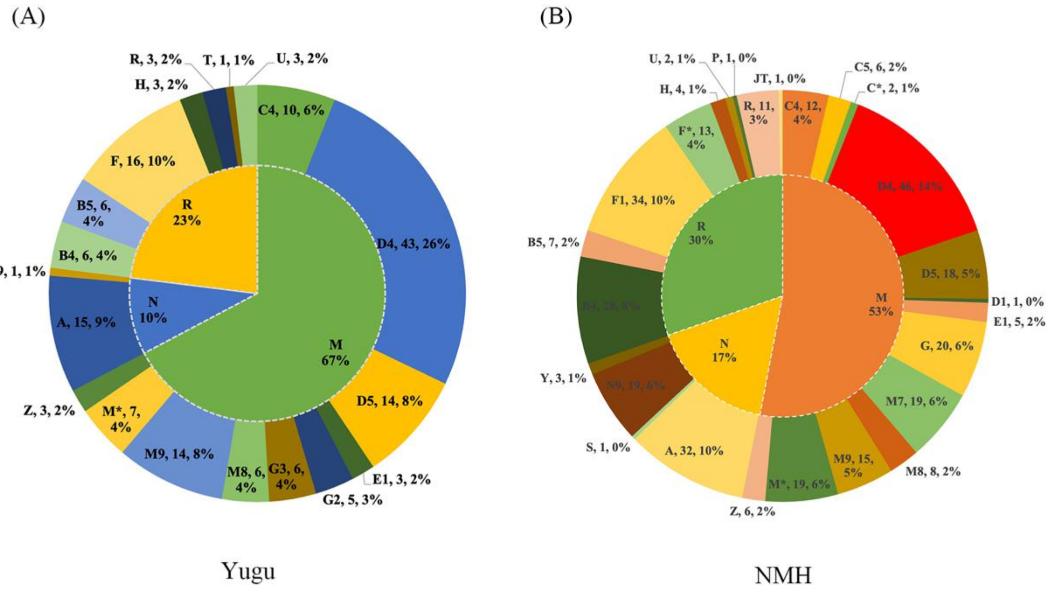

Supplementary Figure S8: (A) Haplogroup assignment based on mtDNA hypervariable regions for Yugu ethnic group; (B) Haplogroup assignment based on mtDNA hypervariable regions for NMH population.

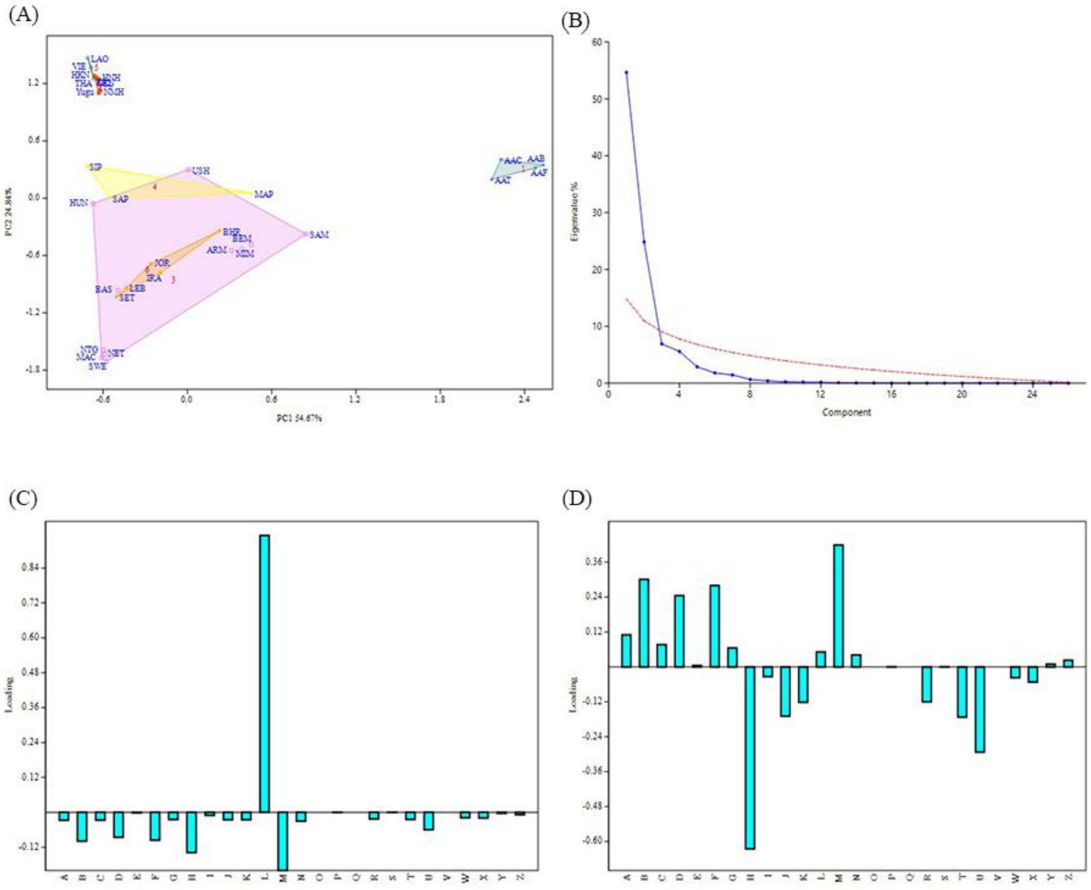

Supplementary Figure S9: PCA conducted based on population haplogroup frequencies. (A):

PCA of the two analyzed populations and the referenced populations with the first two PCs accounted for 79.51% of the total variance; (B): Eigenvalue% of each PC could explain; (C) Variance of each MtDNA haplogroup could explain at PC1 level; (D): Variance of each MtDNA haplogroup could explain at PC2 level.

(A)

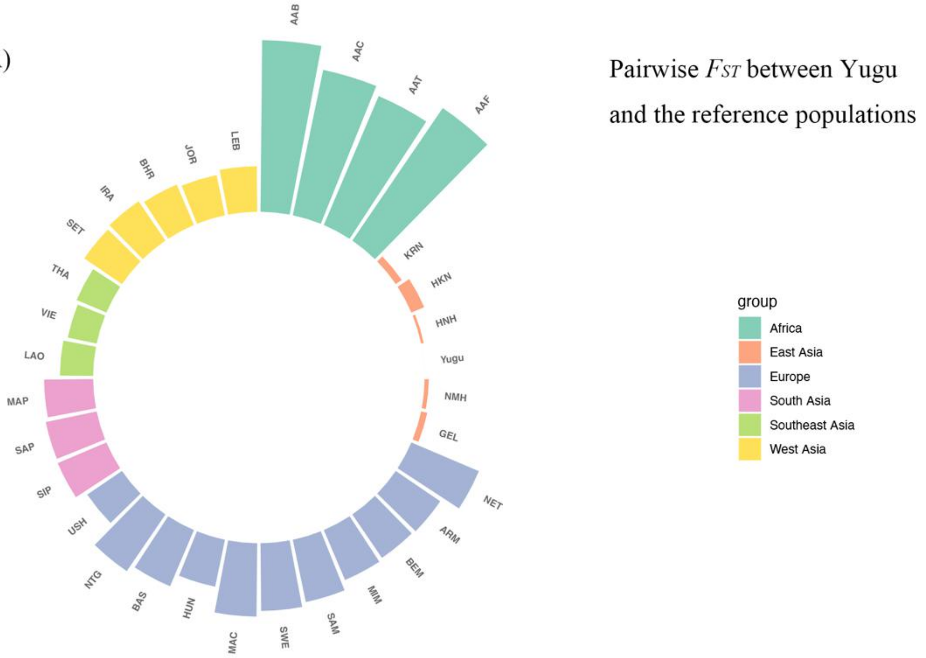

(B)

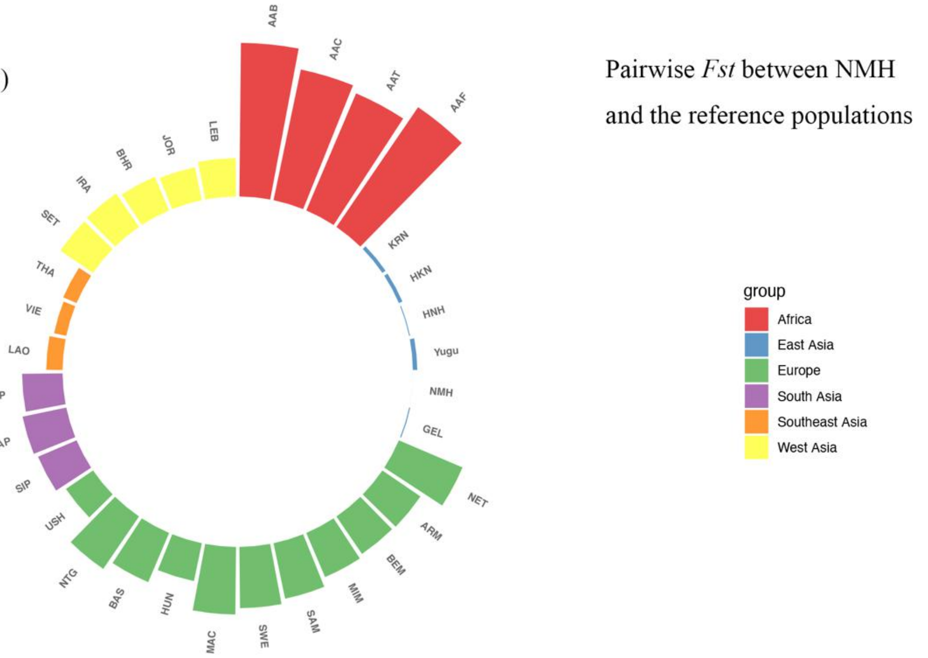

Supplementary Figure S10: (A) Paired F_{ST} values calculated based on haplogroup frequencies between Yugu ethnic group and the reference populations. Different colors correspond to different intercontinental populations, and the height of the bars is proportional to pairwise F_{ST} values; (B) Paired F_{ST} values calculated based on haplogroup frequencies between NMH population and the reference populations. Different colors corresponded to different continental populations, and the height of the bars was proportional to the pairwise F_{ST} values.
